# Quantitative Interactomics in Primary T Cells Provides a Rationale for Concomitant PD-1 and BTLA Coinhibitor Blockade in Cancer Immunotherapy

**DOI:** 10.1016/j.celrep.2019.05.041

**Published:** 2019-06-11

**Authors:** Javier Celis-Gutierrez, Peter Blattmann, Yunhao Zhai, Nicolas Jarmuzynski, Kilian Ruminski, Claude Grégoire, Youcef Ounoughene, Frédéric Fiore, Ruedi Aebersold, Romain Roncagalli, Matthias Gstaiger, Bernard Malissen

**Affiliations:** 1 Centre d’Immunologie de Marseille-Luminy, Aix Marseille Université, INSERM, CNRS, 13288 Marseille, France; 2 Centre d’Immunophénomique, Aix Marseille Université, INSERM, CNRS, 13288 Marseille, France; 3 Department of Biology, Institute of Molecular Systems Biology, ETH Zurich, 8093 Zurich, Switzerland; 4 Faculty of Science, University of Zurich, 8006 Zurich, Switzerland

**Keywords:** T cell, coinhibitory receptors, PD-1, BTLA, protein tyrosine phosphatases, SHP-1, SHP-2, cancer immunotherapy, combination therapy design, quantitative interactomics

## Abstract

Deciphering how TCR signals are modulated by coinhibitory receptors is of fundamental and clinical interest. Using quantitative interactomics, we define the composition and dynamics of the PD-1 and BTLA coinhibitory signalosomes in primary effector T cells and at the T cell-antigen-presenting cell interface. We also solve the existing controversy regarding the role of the SHP-1 and SHP-2 protein-tyrosine phosphatases in mediating PD-1 coinhibition. PD-1 predominantly recruits SHP-2, but when absent, it recruits SHP-1 and remains functional. In contrast, BTLA predominantly recruits SHP-1 and to a lesser extent SHP-2. By separately analyzing the PD-1-SHP-1 and PD-1-SHP-2 complexes, we show that both dampen the TCR and CD28 signaling pathways equally. Therefore, our study illustrates how comparison of coinhibitory receptor signaling via quantitative interactomics in primary T cells unveils their extent of redundancy and provides a rationale for designing combinations of blocking antibodies in cancer immunotherapy on the basis of undisputed modes of action.

## Highlights

• Proteomic profiling of PD-1 and BTLA signalosome assembly in primary T cells
• PD-1 and BTLA show marked quantitative difference in SHP-1 and SHP-2 use
• PD-1 predominantly recruits SHP-2, which can be replaced by SHP-1 when absent
• PD-1 bound to SHP-2 or SHP-1 inhibits both the TCR and CD28 signaling pathways

## Graphical Abstract

**Figure:**
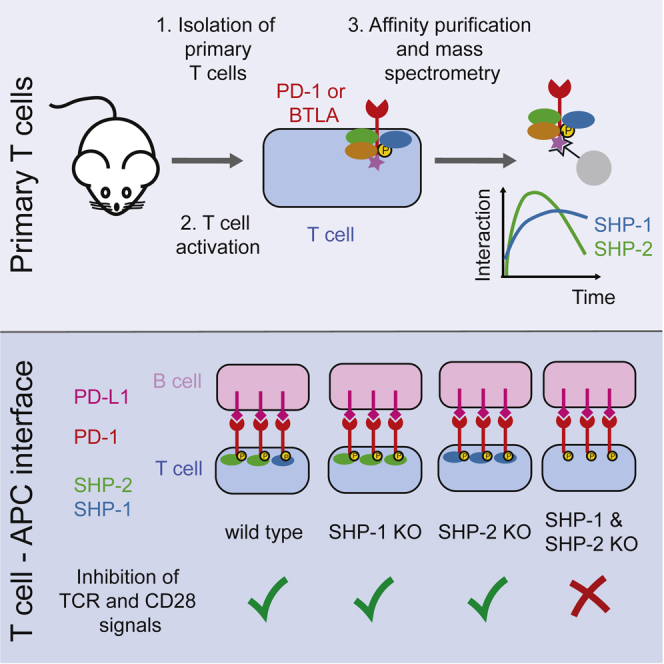

## Teaser

The respective contributions of the SHP-1 and SHP-2 protein-tyrosine phosphatases to PD-1 coinhibition remain debated. Using quantitative interactomics, Celis-Gutierrez et al. define the composition of the PD-1 signalosomes in primary T cells. Deconstructing it into PD-1-SHP-2 and PD-1-SHP-1 complexes showed that they target the TCR and CD28 pathways equally.

## Introduction

Although the T cell antigen receptor (TCR) occupies a central place in T cell activation, it does not work in isolation and is tuned by costimulatory and coinhibitory receptors that report on the immunogenic status of antigen-presenting cells (APCs). Proper integration of TCR, costimulatory, and coinhibitory signals is essential for peripheral tolerance induction and the generation of effector and memory T cells. Moreover, during the effector phase of T cell responses, coinhibitory signals prevent overt tissue disruption. We lack, however, a systems-level understanding of the composition and dynamics of the signaling complexes (signalosomes) used by most T cell costimulators and coinhibitors under physiological conditions. Deciphering how TCR signals are dynamically modulated by a wealth of costimulators and coinhibitors is thus a fundamental question in immunology and of considerable clinical interest because blocking coinhibitory signals via therapeutic antibodies (immune-checkpoint inhibitors) has become a standard treatment in cancer immunotherapy (Hashimoto et al., 2018). Upon chronic antigen exposure, tumor-infiltrating effector T cells (TILs) often upregulate several coinhibitors that altogether dampen TCR and costimulatory signals. The reinvigorated anti-tumor immunity resulting from the coincident blockade of the programmed cell death protein-1 (PD-1) and cytotoxic T lymphocyte-associated protein 4 (CTLA-4) has triggered much interest in combination therapies (Sharpe and Pauken, 2018). However, there is an unmet need for the design of combination therapy trials that are informed by an understanding of the signaling pathways used by candidate coinhibitor pairs.

PD-1 (also called CD279 or PDCD1; Ishida et al., 1992) has two known ligands, known as PD-L1 (CD274) and PD-L2 (CD273), and is rapidly expressed during the naive-to-effector T cell transition to protect T cells from overstimulation (Odorizzi et al., 2015). The B and T lymphocyte attenuator (BTLA; also called CD272) is expressed on naive T cells and transiently upregulated upon TCR engagement (Watanabe et al., 2003). BTLA recognizes the herpes virus entry mediator (HVEM), a member of the tumor necrosis factor receptor superfamily, and BTLA-deficient T cells show increased antigen responsiveness (Krieg et al., 2007, Watanabe et al., 2003). The cytoplasmic tail of both PD-1 and BTLA contains an immunoreceptor tyrosine-based inhibition motif (ITIM) followed by an immunoreceptor tyrosine-based switch motif (ITSM) (Riley, 2009). Upon tyrosine phosphorylation, these motifs recruit cytosolic proteins that reduce T cell antigen sensitivity, metabolic reprogramming, and entry into the cell cycle (Sharpe and Pauken, 2018). So far, most studies aiming at identifying such cytosolic proteins have pointed toward the Src homology 2 (SH2) domain-containing protein-tyrosine phosphatases (PTPases) SHP-1 (also called PTPN6) and SHP-2 (also called PTPN11) (Riley, 2009). Those studies relied on overexpression and thus remained qualitative. They also failed to determine whether SHP-1 and SHP-2, which are structurally related and coexpressed in T cells, act in a redundant manner to mediate PD-1 coinhibition under physiological conditions, a key issue in view of a recent study suggesting that SHP-2 is dispensable for PD-1 coinhibition *in vivo* (Rota et al., 2018).

Redundant molecules can compensate for each other’s loss by taking over and performing the exact same function. Biological redundancy is frequently associated with pairs of genes that derive from the same ancestral gene (known as paralogs). Among coinhibitors, PD-1 and BTLA are evolutionary related (Riley, 2009) and coexpressed on human and mouse tumor-antigen specific CD8^+^ T cells (Ahrends et al., 2017, Baitsch et al., 2012). Accordingly, BTLA can likely substitute for PD-1 in conditions in which immune-checkpoint inhibitors target PD-1, and *in vitro* studies support that view (Derré et al., 2010, Fourcade et al., 2012). By using gene-edited mice that permit affinity purification coupled with mass spectrometry (AP-MS) analysis, we defined the composition, stoichiometry, and dynamics of the PD-1 and BTLA signalosomes in primary T cells. Moreover, we solved the existing inconsistency regarding the respective role of SHP-1 and SHP-2 in mediating PD-1 coinhibition.

## Results

### The PD-1 Signalosome of Primary Effector CD4^+^ T Cells

**Figure 1:**
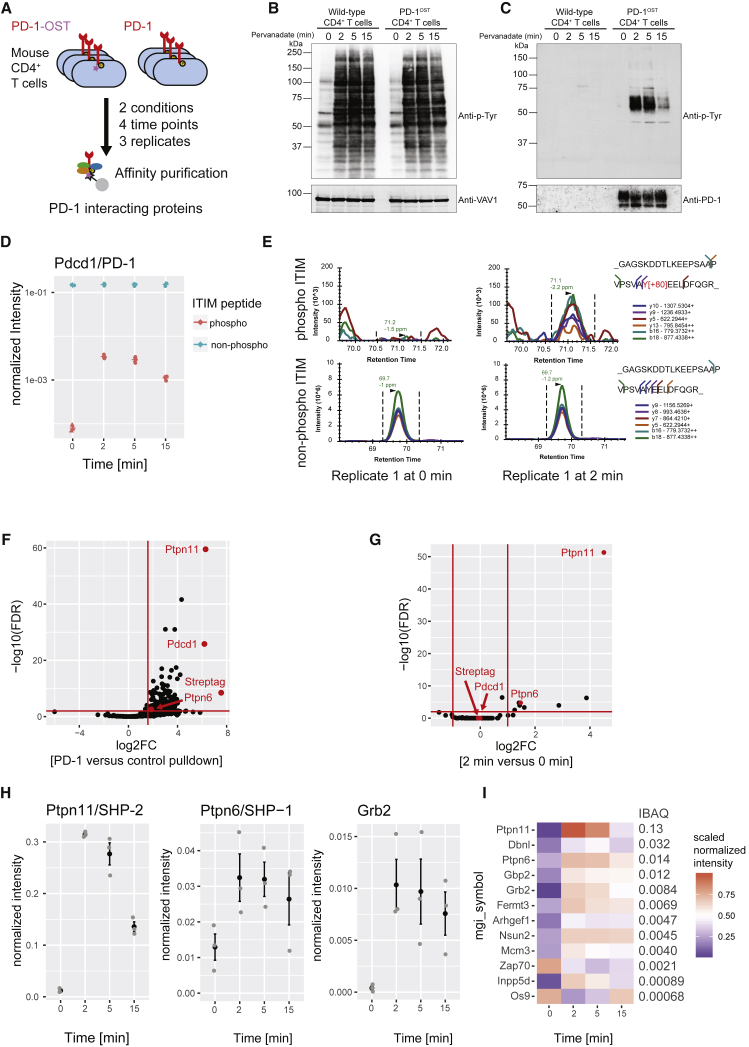
Composition and Dynamics of the PD-1 Signalosome in Primary CD4^+^ T Cells (A) Overview of AP-MS analysis of PD-1^+^ CD4^+^ effector T cells from WT mice (PD-1) and gene-edited mice expressing endogenous PD-1 molecules tagged with a Twin-Strep-tag (PD-1^OST^). T cells were lysed prior to or after stimulation for 2, 5, and 10 min with pervanadate followed by affinity purification of PD-1-OST protein complexes. (B) Immunoblot analysis of equal amounts of proteins from total lysates of PD-1^+^ CD4^+^ effector T cells from WT and PD-1^OST^ mice left unstimulated (0) or stimulated with pervanadate for 2, 5, and 15 min and probed with antibody to phosphorylated tyrosine (Anti-p-Tyr) or anti-VAV1 (loading control). (C) Immunoblot analysis of equal amounts of proteins from total lysates of cells as in (B), subjected to affinity purification on Strep-Tactin-Sepharose beads, followed by elution of proteins with D-biotin, and probed with antibody to phosphorylated tyrosine (Anti-p-Tyr) or anti-PD-1 (affinity purification control). Left margins of (B) and (C), molecular size in kilodaltons. In (B) and (C), data are representative of three independent experiments. (D) Intensity values for the non-phosphorylated and phosphorylated forms of the peptide containing the PD-1 ITIM motif. Values were normalized to that of the PD-1 bait (see STAR Methods). (E) Elution profile of the six different fragment ions from the phosphorylated and non-phosphorylated forms of the ITIM-containing peptide measured at 0 and 2 min after pervanadate treatment. (F) Volcano plot showing proteins significantly enriched in CD4^+^ T cells expressing PD-1-OST molecules compared with control CD4^+^ T cells expressing similar levels of untagged PD-1 molecules at 2 min after pervanadate treatment. (G) Volcano plot showing proteins significantly enriched in CD4^+^ T cells expressing PD-1-OST molecules 2 min after pervanadate treatment compared with untreated CD4^+^ T cells. In (F) and (G), the PD-1/PDCD1, SHP-2/PTPN11, SHP-1/PTPN6 proteins, and Twin-Strep-tag peptide (Streptag) are shown in red, and the x and y axes show the average fold change (log_2_FC) in protein abundance and the statistical significance, respectively (see STAR Methods). (H) Intensity for selected interactors across the conditions and replicates. Mean ± SEM is depicted (black), and individual values are shown as gray dots. (I) Heatmap depicting the average intensity of PD-1 interactors across time points (row-normalized to the maximal value). The iBAQ column shows the stoichiometry of interaction of each prey with the PD-1-OST bait 2 min after pervanadate treatment.

To identify the proteins that interact with PD-1 in primary effector T cells, we generated mice expressing a Twin-Strep-tag (OST) for affinity purification at the C terminus of endogenous PD-1 proteins (PD-1^OST^ mice) (Figure 1A). T cells with normal phenotype and numbers were present in PD-1^OST^ mice (Figure S1A). Following stimulation for 3.5 days with anti-TCR and anti-CD28 antibodies, they expressed levels of PD-1 comparable with their wild-type (WT) counterparts (Figure S1B). Purified CD4^+^ T cells from WT and PD-1^OST^ mice responded similarly to stimulation with anti-TCR and anti-CD28 antibodies (Figure S1C). After activation for 2, 5, and 15 min with pervanadate (Figure 1B), a surrogate for TCR stimulation that triggers maximal phosphorylation of the PD-1 ITSM and ITIM motifs (Watanabe et al., 2003), PD-1^OST^ and WT CD4^+^ T cells showed a similar pattern of inducible tyrosine phosphorylation. As expected, PD-1-OST molecules were only affinity purified from PD-1^OST^ samples (Figure 1C, bottom panel) and showed a transient increase in tyrosine phosphorylation, peaking 2 min after pervanadate stimulation and leading to their binding with tyrosine-phosphorylated species (Figure 1C, top panel).

To identify and quantify the proteins (the “prey”) that associate with PD-1-OST molecules (the “bait”) over the course of pervanadate stimulation, we applied our SWATH (sequential window acquisition of all theoretical fragment ion spectra)-MS workflow (Caron et al., 2017; STAR Methods) to PD-1^OST^ CD4^+^ T cells that were briefly expanded to induce high levels of PD-1 and then treated for 2, 5, or 15 min with pervanadate or left untreated. To eliminate non-specific contaminants, control experiments were performed using briefly expanded WT CD4^+^ T cells expressing similar levels of untagged PD-1 molecules. For each time point, three independent biological replicates were performed, and the copurified proteins (the “interactome”) were compared using SWATH-MS (Figure 1A). As expected and consistent with immunoblot analysis (Figure 1C), an increase in phosphorylation of tyrosine 225 of the PD-1 ITIM occurred after pervanadate treatment (Figures 1D and 1E; Table S1). To identify proteins with a putative role in PD-1 coinhibition, we applied two orthogonal filters. In the first step, we identified 219 proteins that showed a more than 3-fold increase in abundance in purifications from PD-1^OST^ CD4^+^ T cells compared with WT CD4^+^ T cells (Figure 1F; Table S2). In the second filtering step, we identified 12 interactors that changed in abundance at least 2-fold following pervanadate treatment (Figure 1G; Table S2). The final list included the known PD-1 interactors SHP-1, SHP-2, and GRB2, which showed maximal association 2 min after pervanadate treatment (Figure 1H). Candidate interactors with known (DBNL, FERMT3, ZAP70, and INPP5D/SHIP1) or unrecognized (ARHGEF1, OS9, GBP2, NSUN2, and MCM3) links to T cell signaling were also identified (Figure 1I).

For each of the identified PD-1-prey interactions, we estimated the relative quantity of prey bound to PD-1 (a value known as the interaction stoichiometry) by intensity-based absolute quantification (iBAQ; Schwanhäusser et al., 2011) and the estimated values were normalized to the bait (Figure 1I, iBAQ column; Table S3). Two minutes after pervanadate treatment, SHP-2 was by far the most abundant PD-1 interactor, occupying 13% of the available PD-1-OST molecules, whereas SHP-1 and GRB2 reached 9 and 15 times lower interaction stoichiometries, respectively. Most of the additional candidate interactors showed interaction stoichiometries lower than that of GRB2. Thus SHP-2 was the most abundant interactor that dynamically associated with PD-1 following pervanadate stimulation in primary CD4^+^ T cells.

### The BTLA Signalosome of Primary Effector CD4^+^ T Cells

The PD-1 and BTLA cytoplasmic segments possess a similar exon-intron organization that encodes a pair of immunoreceptor tyrosine-based motifs and differ from those of the CD28, CTLA4, and ICOS molecules (Figure S2A; Riley, 2009). To determine whether the evolutionary relationship existing between PD-1 and BTLA translates into the assembly of similar signalosomes, mice expressing an OST at the C terminus of endogenous BTLA proteins (BTLA^OST^ mice) were developed. They contained T cells with similar phenotype and numbers compared with WT mice (Figure S2B) and that expressed similar levels of BTLA molecules 3 days after stimulation with anti-CD3 and anti-CD28 antibodies (Figure S2C). Purified CD4^+^ T cells from BTLA^OST^ mice proliferated similarly to their WT counterparts upon stimulation with anti-TCR and anti-CD28 antibodies (Figure S2D). Consistent with the presence of five N-glycosylation sites in BTLA, treatment of affinity-purified BTLA-OST molecules with glycopeptidase F (PNGase F) converted the native BTLA-OST band into two bands (Figure S2E). This differed from PD-1, in which PNGase F treatment converted the native N-glycosylated PD-1-OST molecules into a single band corresponding to the molecular weight expected for unmodified PD-1-OST polypeptides (Figure S1D). The unexpected upper band found in BTLA digest suggests that a fraction of BTLA molecules undergoes additional post-translational modifications such as palmitoylation, a possibility consistent with the presence of a conserved cysteine at the junction of the BTLA transmembrane and cytoplasmic segments and supported by a proteomic study of a lymphoblastic cell line (Colquhoun et al., 2015).

**Figure 2:**
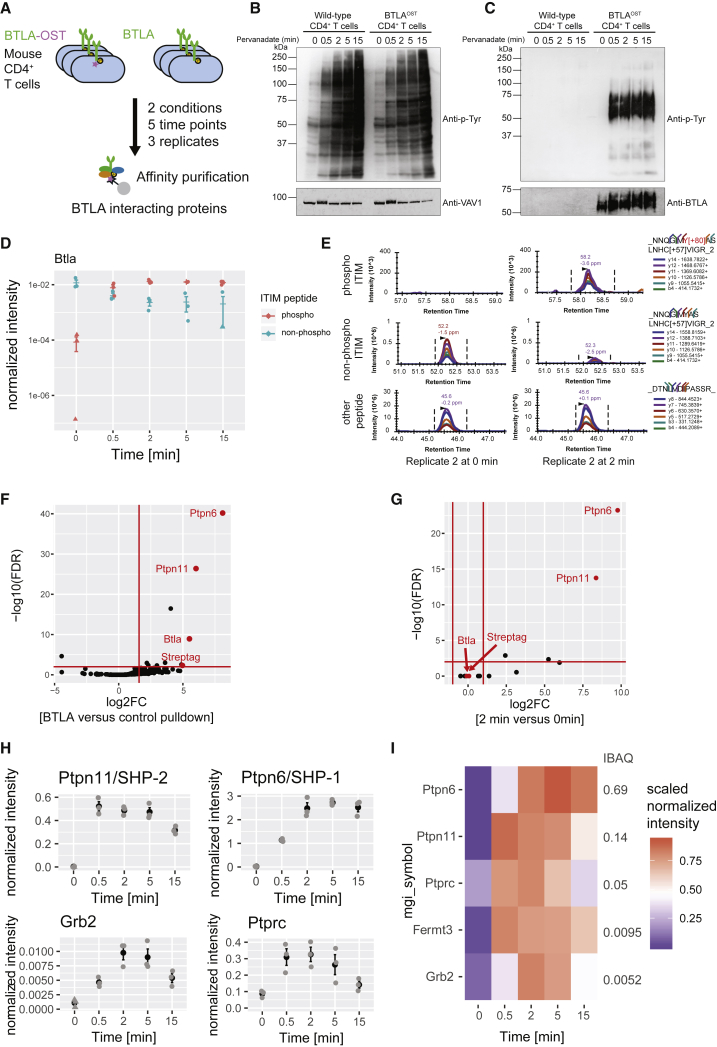
Composition and Dynamics of the BTLA Signalosome in Primary CD4^+^ T Cells (A) Overview of AP-MS analysis of BTLA^+^ CD4^+^ effector T cells isolated from WT mice (BTLA) and gene-edited mice expressing endogenous BTLA molecules tagged with a Twin-Strep-tag (BTLA^OST^). T cells were lysed prior to or after stimulation for 0.5, 2, 5, and 15 min with pervanadate followed by affinity purification of BTLA-OST protein complexes. (B) Immunoblot analysis of equal amounts of proteins from total lysates of WT and BTLA^OST^ CD4^+^ T cells left unstimulated (0) or stimulated for 0.5, 2, 5, and 15 min with pervanadate and probed with antibody specific for phosphorylated tyrosine (Anti-p-Tyr) or VAV1 (loading control). (C) Immunoblot analysis of equal amounts of proteins from total lysates of cells as in (B), subjected to affinity purification on Strep-Tactin-Sepharose beads, followed by elution of proteins with D-biotin, and probed with antibody specific for phosphorylated tyrosine (Anti-p-Tyr) or BTLA (affinity purification control). Left margins of (B) and (C), molecular size in kilodaltons. Data in (B) and (C) are representative of three independent experiments. (D) Intensity values for the non-phosphorylated and phosphorylated forms of the peptide containing the BTLA ITIM motif. Values were normalized to that of the BTLA bait, and one value has been imputed (triangle). (E) Elution profile of the six different fragment ions from the phosphorylated and non-phosphorylated form of the ITIM-containing peptide measured at 0 and 2 min after pervanadate treatment. (F) Volcano plot showing proteins significantly enriched in CD4^+^ T cells expressing BTLA-OST molecules compared with control CD4^+^ T cells expressing similar levels of untagged BTLA proteins at 2 min after pervanadate treatment. (G) Volcano plot showing proteins significantly enriched in CD4^+^ T cells expressing BTLA-OST molecules 2 min after pervanadate treatment compared with untreated cells. In (F) and (G), the BTLA, SHP-2/PTPN11, SHP-1/PTPN6 proteins, and Twin-Strep-tag peptide (Streptag) are shown in red, and the x and y axes show the average fold change (log_2_FC) in protein abundance and the statistical significance, respectively. (H) Intensity for selected interactors across the different replicates. Mean ± SEM is depicted (black), and individual values are shown as gray dots. (I) Heatmap depicting BTLA interactor intensity across time points (row-normalized to the maximal value). The iBAQ column shows the stoichiometry of interaction of each prey with the BTLA bait 2 min after pervanadate treatment.

Briefly expanded primary CD4^+^ T cells from BTLA^OST^ and WT mice were lysed prior to or after a further activation for 0.5, 2, 5, and 15 min with pervanadate to trigger maximal phosphorylation of the BTLA ITIM and ITSM motifs (Gavrieli et al., 2003; Figure 2A). CD4^+^ T cells from WT and BTLA^OST^ mice showed a similar global pattern of pervanadate-induced tyrosine phosphorylation (Figure 2B). BTLA-OST molecules purified from BTLA-1^OST^ samples were tyrosine phosphorylated upon pervanadate treatment (Figure 2C), and AP-MS confirmed that tyrosine 274 of the BTLA ITIM was phosphorylated upon pervanadate treatment (Figures 2D and 2E; Table S1). The coincident decrease observed in the intensity of the non-phosphorylated ITIM peptide showed that a majority of BTLA molecules was phosphorylated (Figure 2E; Table S1).

By applying the same filtering strategy used for PD-1, we identified five high-confidence BTLA interactors (SHP-1, SHP-2, GRB2, PTPRC, and FERMT3; Figures 2F and 2G; Table S2). They all showed maximal association with BTLA-OST molecules between 2 and 5 min after pervanadate treatment (Figures 2H and 2I; Table S2). Using iBAQ intensities and considering that SH2 domain-containing PTPase binds with a one-to-one stoichiometry to both PD-1 and BTLA, we were able to compare the relative abundance of SHP-1 and SHP-2 associated to PD-1 (Figure 1I; Table S3) and BTLA (Figure 2I; Table S3). Two minutes after pervanadate treatment, comparable percentages of the available PD-1-OST (13%) and BTLA-OST (14%) molecules were associated with SHP-2. In marked contrast, 69% and 1.4% of the BTLA-OST and PD-1-OST molecules were associated with SHP-1, respectively. Therefore, PD-1 showed a 10-fold preference for SHP-2 over SHP-1 and BTLA a 5-fold preference for SHP-1 over SHP-2, demonstrating that the evolutionary related PD-1 and BTLA receptors showed quantitative differences in their use of the two members of the SH2 domain-containing PTPase family.

### A Model Permitting PD-1 Interactomics at the T Cell-APC Interface

**Figure 3:**
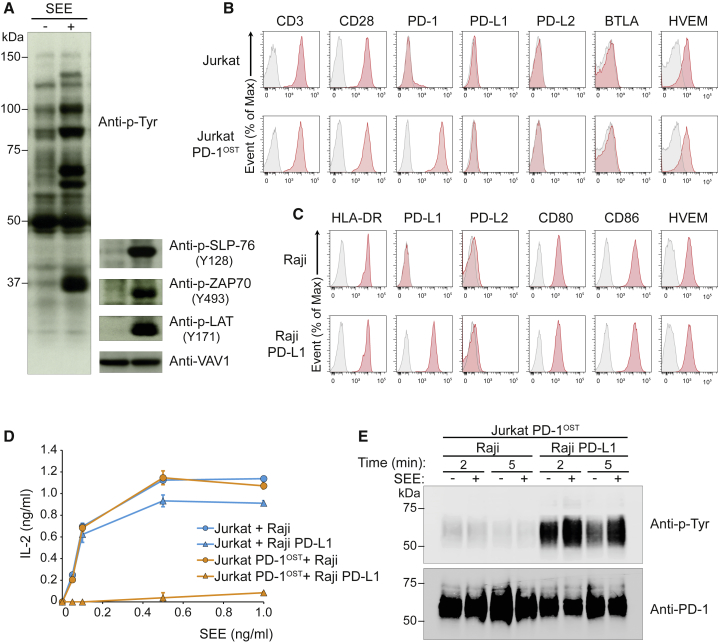
Outcome of PD-1-PD-L1 Engagement at T Cell-APC Interface (A) Jurkat-PD-1^OST^ cells were stimulated with Raji cells that have been preincubated in the absence (−) or presence (+) of 200 ng/mL SEE and lysed 2 min after the initiation of cell-cell contact. Immunoblot analysis of equal amounts of lysates from the specified conditions probed with antibody to phosphorylated proteins (Anti-p-Tyr) or with phospho-tyrosine-specific antibodies directed against SLP76 pY128, ZAP70 pY493, LAT pY171 or VAV1 (loading control). Left margin, molecular size in kilodaltons (kDa). Data are representative of three independent experiments. (B) Expression of CD3, CD28, PD-1, PD-L1, PD-L2, BTLA, and HVEM at the surface of Jurkat cells and Jurkat-PD-1^OST^ cells, analyzed using flow cytometry. (C) Expression of HLA-DR, PD-L1, PD-L2, CD80, CD86, and HVEM at the surface of Raji cells and Raji-PD-L1 cells, analyzed using flow cytometry. In (B) and (C), gray shaded curves correspond to isotype-matched control antibody (negative control), and data are representative of two independent experiments. (D) IL-2 production by Jurkat and Jurkat-PD-1^OST^ cells stimulated for 24 h with either Raji or Raji-PD-L1 cells in the absence (0) or presence of the specified amounts of SEE. Data are representative of three independent experiments, and mean and SEM are shown. (E) Jurkat-PD-1^OST^ cells stimulated at 37°C with Raji or Raji-PD-L1 cells preincubated in the absence (–) or presence (+) of 200 ng/mL SEE and lysed 2 and 5 min after the initiation of cell-cell contact. Immunoblot analysis of equal amounts of lysates from the specified conditions subjected to affinity purification on Strep-Tactin-Sepharose beads and probed with antibody to phosphorylated proteins (Anti-p-Tyr). Also shown are loading controls probed with anti-PD-1 antibody. Left margin, molecular size in kilodaltons (kDa). Data are representative of two independent experiments.

To determine the composition, stoichiometry, and dynamics of the PD-1 interactome that assembles following physiological T cell-APC interaction, we used a model in which Jurkat human leukemic T cells were stimulated with Raji human lymphoblastoid B cells presenting the superantigen staphylococcal enterotoxin E (Raji + SEE). Upon stimulation, Jurkat T cells contained large numbers of tyrosine-phosphorylated species (Figure 3A) and produced IL-2 in a TCR- and CD28-dependent manner (Tian et al., 2015). Jurkat cells expressed minute levels of PD-1 (Figure 3B) and were transfected with human PD-1 molecules tagged with an OST at their C terminus (Jurkat-PD-1^OST^; Figure 3B). Raji cells lacked PD-L1 and PD-L2 and were transfected with human PD-L1 molecules (Raji-PD-L1; Figure 3C). PD-L1 can interact with CD80 in *cis* at the surface of APCs and prevent it from binding to T cell-expressed PD-1 (Sugiura et al., 2019). However, the levels of PD-L1 expression achieved on Raji-PD-L1 cells overcame the inhibitory interactions imposed by the pool of CD80 molecules expressed on Raji cells and permitted the remaining “free” PD-L1 molecules to mediate PD-1 coinhibition (Figures 3C and 3D). Note that contrary to primary T cells, Jurkat T cells express neither PD-L1 nor PD-L2 on their surface (Figure 3B). Therefore, the occurrence of PD-1-PD-L1 interactions in our model is due only to T cell-APC interactions and not to T cell-T cell or *cis* interactions between PD-1 and PD-L1/2 (Zhao et al., 2018). Moreover, the lack of BTLA on Jurkat T cells prevented it from contributing to the observed phenotype (Figure 3B).

Jurkat-PD-1^OST^ cells stimulated with Raji + SEE produced IL-2 in amounts comparable with those reached when Jurkat cells were stimulated with Raji + SEE (Figure 3D). Likewise, stimulation of Jurkat cells in the presence of Raji + SEE or of Raji-PD-L1 + SEE resulted in similar levels of IL-2 production (Figure 3D). In marked contrast, stimulation of Jurkat-PD-1^OST^ cells with Raji-PD-L1 + SEE totally ablated IL-2 production (Figure 3D). Interaction of Jurkat-PD-1^OST^ cells with Raji cells with or without SEE induced barely detectable levels of PD-1 phosphorylation (Figure 3E). Maximal PD-1 phosphorylation required co-engagement of both the TCR and PD-1 and was reached 2 min after initiation of T cell-APC contact (Figure 3E). In the sole presence of PD-1-PD-L1 engagement, PD-1 phosphorylation also occurred, although with a lower intensity (Figure 3E). Therefore, by rigorously controlling that PD-1-PD-L1 engagement occurred in “*trans*,” our T cell-APC-based model enables appropriate analysis of PD-1 signalosome formation at a T cell-APC interface.

### Characterization of the Human PD-1 Signalosome at the T Cell-APC Interface

**Figure 4:**
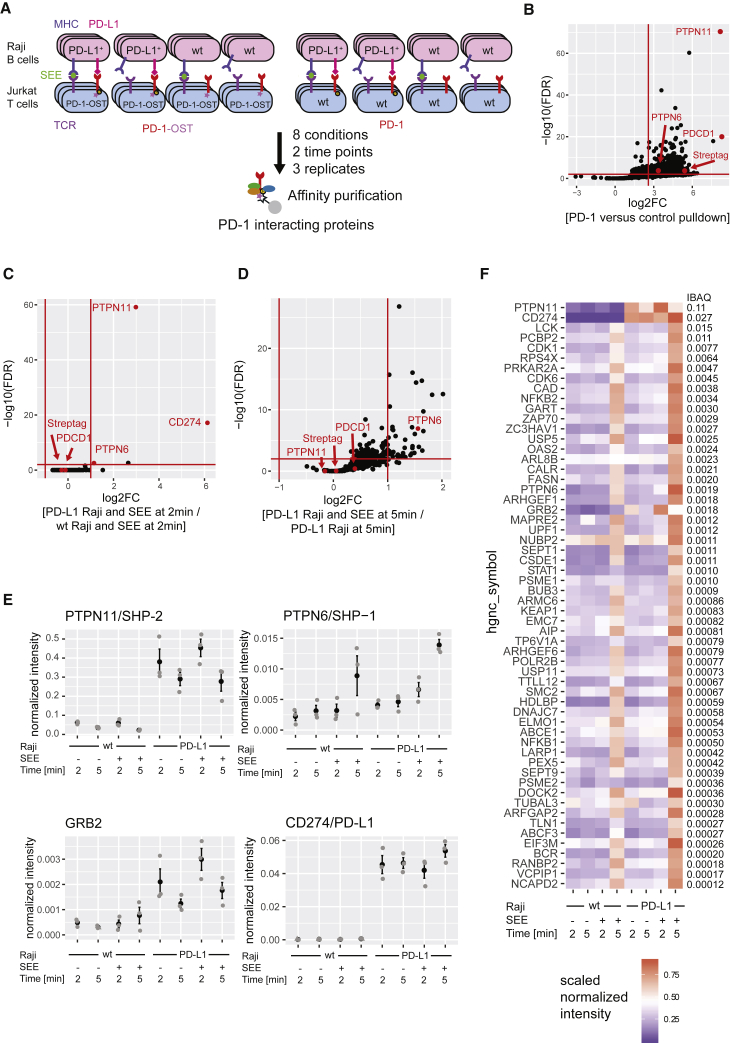
Composition and Dynamics of the Human PD-1 Interactome following Physiological T Cell-APC Interaction (A) Overview of AP-MS analysis of PD-1 and PD-1-OST expressing Jurkat T cells stimulated with Raji B cells expressing or not PD-L1 and in the presence or absence of SEE. The various T cell-B cell pellets were lysed after been kept in contact for 2 and 5 min, followed by affinity purification of PD-1-OST protein complexes. (B) Volcano plot showing proteins significantly enriched in Jurkat-PD-1^OST^ cells compared with Jurkat cells expressing similar levels of untagged PD-1 proteins at 2 min after stimulation with Raji-PD-L1 cells + SEE. (C) Volcano plot showing proteins significantly enriched in Jurkat-PD-1^OST^ cells 2 min after stimulation with Raji-PD-L1 cells + SEE compared with Jurkat-PD-1^OST^ cells stimulated for 2 min with Raji cells + SEE. (D) Volcano plot showing proteins significantly enriched in Jurkat-PD-1^OST^ cells 5 min after stimulation with Raji-PD-L1 cells + SEE compared with Raji-PD-L1 cells without SEE stimulation. In (B), (C), and (D), the PD-1/PDCD1, PD-L1/CD274, SHP-2/PTPN11, SHP-1/PTPN6 proteins, and Twin-Strep-tag peptide (Streptag) are shown in red, and the x and y axes show the average fold change (log_2_FC) in protein abundance and the statistical significance, respectively. (E) Intensity for selected interactors across the different replicates. Mean ± SEM is depicted (black), and individual values are shown as gray dots. (F) Heatmap depicting PD-1 interactor intensity across time points (row-normalized to the maximal value). The iBAQ column shows the stoichiometry of interaction of each prey with the PD-1 bait at 2 min after stimulation with Raji-PD-L1 cells + SEE.

Raji-PD-L1 cells were preincubated in the presence or absence of SEE and mixed together with Jurkat-PD-1^OST^ T cells in microcentrifuge tubes. After a quick centrifugation to promote cell-cell contact, pellets were kept at 37°C for 2 and 5 min, immediately lysed, and subjected to AP-SWATH-MS. To identify non-specific contaminants, control experiments were performed using Jurkat T cells expressing levels of untagged PD-1 molecules comparable with Jurkat-PD-1^OST^ T cells (Figure 4A). Raji cells with or without SEE were also used as control APC. Three independent biological replicates were run for the two time points of the eight conditions and subjected to AP-SWATH-MS. We applied again the two-step filtering described above and after selecting proteins specifically interacting with the bait (fold change larger than 6 with a false discovery rate [FDR] < 0.01 in three biological replicates; Tables S1 and S2), we selected those interactors that significantly changed in abundance following TCR and/or PD-1-PD-L1 engagement (Figures 4B–4D; see STAR Methods). Across all conditions, 58 PD-1-interacting proteins passed these filter criteria (Figure 4F; Table S2). When Raji-PD-L1 cells were used as APCs, PD-L1 was present in the PD-1 interactome regardless of TCR engagement (Figure 4E), a finding congruent with its micromolar affinity for PD-1 (Li et al., 2017). We also confirmed the presence of five PD-1 interactors (GRB2, ARHGEF1, SHP-1, ZAP70, and SHP-2), which are the human orthologs of the interactors we already identified in PD-1-OST complexes purified from pervanadate-treated mouse CD4^+^ T cells (Table S2). SHP-2 was by far the most abundant PD-1 interactor, occupying 11% of the available PD-1-OST molecules 2 min after T cell-APC contact, whereas SHP-1 and GRB2 reached 57 and 61 times lower interaction stoichiometries, respectively (Figure 4F). SHP-2 and GRB2 were also capable of associating with PD-1 in the sole presence of PD-1-PD-L1 engagement, an association that was further enhanced after 2 min of TCR activation (Figure 4E). Whereas the binding of SHP-2 and GRB2 to PD-1 peaked at 2 min of T-APC contact (Figure 4F), a sizable number of the low-stoichiometry interactors recruited by PD-1 (including SHP-1) showed increased binding 5 min after T-APC contact (for details see Figure S3). Such a late increase in interactome complexity was not observed following pervanadate stimulation (Figure 1I) or stimulation of Jurkat-PD-1^OST^ T cells with Raji + SEE and might thus correspond to the association of those PD-1-OST molecules that are engaged at the Jurkat-PD-1^OST^-Raji-PD-L1 + SEE interface with the molecular machinery responsible for their endocytosis. Therefore, akin to the situation observed in primary effector CD4^+^ T cells activated with pervanadate, the PD-1 interactome observed 2 min after the onset of T-APC interaction was quantitatively dominated by SHP-2 (Table S3).

### T Cell Origin of the Components of the PD-1 Signalosome that Forms at the T-APC Interface

Following stimulation of Jurkat-PD-1^OST^ cells with Raji-PD-L1 (Figure 4A), the resulting cell pellets were lysed and subjected to AP-MS. To exclude the possibility that part of the documented PD-1 signalosome assembled post-lysis from proteins originating from Raji-PD-L1 cells, we used stable isotope labeling by amino acids in cell culture (SILAC) to label Raji-PD-L1 cells with lysine and arginine containing heavy isotopes. After incubating Jurkat-PD-1^OST^ and Raji-PD-L1 cells for 2 min in the presence of SEE and lysing them, this differential labeling approach should allow to distinguish “heavy” peptides derived from Raji-PD-L1 cells from unlabeled (“light”) peptides derived from Jurkat-PD-1^OST^ cells. As expected, all the peptides corresponding to PD-1-OST molecules were exclusively of Jurkat-PD-1^OST^ origin, whereas PD-L1 peptides derived exclusively from Raji-PD-L1 cells (Figure S4). Considering that the Raji-PD-L1 and control Raji cells used in SILAC experiments have been rendered SHP-2^–^ via CRISPR-Cas9 editing (see below), SHP-2 was exclusively of Jurkat-PD-1^OST^ origin and remained the most abundant PD-1 interactor (Figure S4). Interestingly, SHP-1 and GRB2 showed a mixed origin, suggesting that during the 1.5 h affinity purification, some exchange occurred between the SHP-1 and GRB2 molecules of Jurkat-PD-1^OST^ origin that were bound to PD-1 at the time of lysis and incoming SHP-1 and GRB2 molecules of Raji-PD-L1 origin (Figure S4). This finding is congruent with photobleaching studies showing that although TCR-triggered signalosomes persist for minutes, some of their components, such as the ZAP-70 protein-tyrosine kinase, continuously dissociate (dwell time of ∼10 s) and are replaced by free ZAP-70 molecules present in the cytosol (Bunnell et al., 2002, Taylor et al., 2017). Therefore, SILAC labeling of Raji cells showed that under our experimental conditions, the major components of the PD-1 signalosomes that form at the T-APC interface are comparable with those observed using unlabeled Raji cells and originate either exclusively (SHP-2) or in large part (SHP-1 and GRB2) from Jurkat-PD-1^OST^ T cells.

### SHP-1 Can Fully Replace SHP-2 for PD-1 Coinhibition in Jurkat T Cells

**Figure 5:**
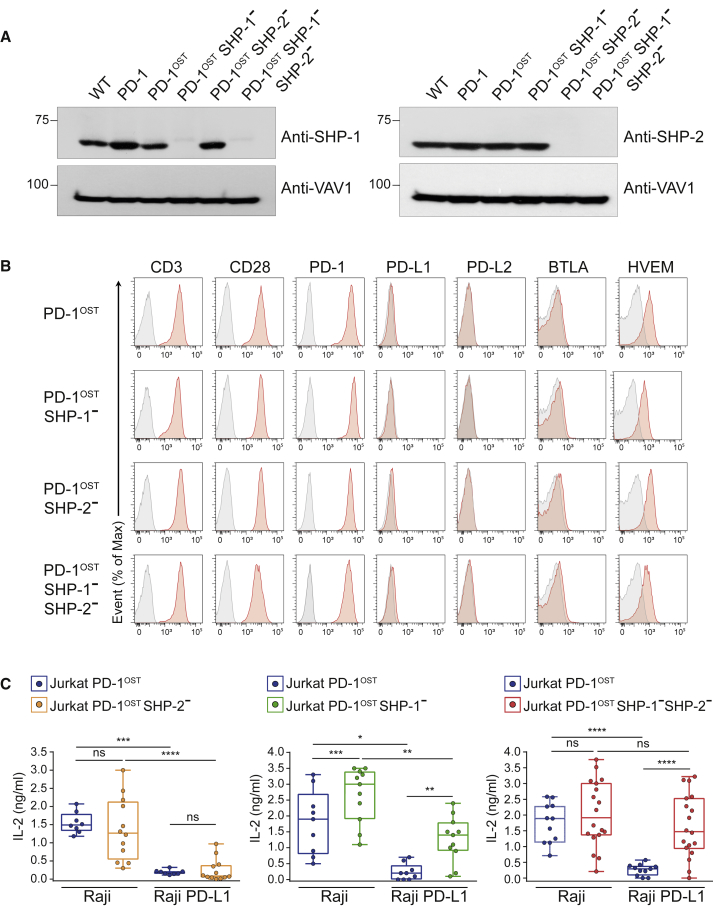
Either SHP-1 or SHP-2 Suffices for PD-1 Coinhibition in Jurkat T Cells (A) Immunoblot analysis of equal amounts of proteins from total lysates of representative clones of WT Jurkat, Jurkat-PD-1, Jurkat-PD-1^OST^, Jurkat-PD-1^OST^ SHP-1^–^, Jurkat-PD-1^OST^ SHP-2^–^, and Jurkat-PD-1^OST^ SHP-1^–^SHP-2^–^ cells probed with antibodies to SHP-1, SHP-2, or VAV1 (loading control). Left margin, molecular size in kilodaltons. Data are representative of two independent experiments. (B) Jurkat-PD-1^OST^, Jurkat-PD-1^OST^ SHP-1^–^, Jurkat-PD-1^OST^ SHP-2^–^, and Jurkat-PD-1^OST^ SHP-1^–^ SHP-2^–^ cells were analyzed using flow cytometry for expression of CD3, CD28, PD-1, PD-L1, PD-L2, BTLA, and HVEM. Gray shaded curves correspond to isotype-matched control antibody (negative control), and data are representative of two independent experiments. (C) IL-2 production by Jurkat-PD-1^OST^, Jurkat-PD-1^OST^ SHP-1^–^, Jurkat-PD-1^OST^ SHP-2^–^, and Jurkat-PD-1^OST^ SHP-1^–^SHP-2^–^ clones stimulated with Raji cells or Raji-PD-L1 cells in the presence of 1 ng/mL SEE. Analysis of IL-2 production was performed 24 h after stimulation. Expression of IL-2 (ng/mL) using boxplot with the median, boxed interquartile range, and whiskers extending to the most extreme point up to 1.5 times the interquartile range. Each dot corresponds to a clone of the specified Jurkat cells. ^∗^p ≤ 0.05, ^∗∗^p ≤ 0.01, ^∗∗∗^p ≤ 0.001, and ^∗∗∗∗^p ≤ 0.0001; ns, non-significant (unpaired Student’s t test). Data are representative of two independent experiments.

The AP-SWATH-MS experiments showed that PD-1-SHP-2 complexes are 9 and 57 times more abundant than PD-1-SHP-1 complexes after activation with pervanadate and APC, respectively (Figures 1 and 4). To test whether PD-1-SHP-2 and PD-1-SHP-1 complexes can both contribute to PD-1 coinhibition, clones of Jurkat-PD-1^OST^ cells deprived of SHP-1 (Jurkat-PD-1^OST^ SHP-1^–^), SHP-2 (Jurkat-PD-1^OST^ SHP-2^–^), or both SHP-1 and SHP-2 (Jurkat-PD-1^OST^ SHP-1^–^ SHP-2^–^) were developed using CRISPR-Cas9 gene editing. They were validated using SHP-1- and SHP-2-specific antibodies (Figure 5A) and shown to express levels of CD3, CD28, PD-1, PD-L1, PD-L2, BTLA, and HVEM similar to the parental Jurkat-PD-1^OST^ (Figure 5B). Because clones of the Jurkat T cell line often showed variation in the levels of IL-2 they produced in response to TCR + CD28 stimulation (Bray et al., 2018), we systematically analyzed for each condition up to ten independent clones.

Comparison of IL-2 production by Jurkat-PD-1^OST^ and Jurkat-PD-1^OST^ SHP-2^–^ clones in response to Raji + SEE showed that in the absence of PD-1-PD-L1 engagement, the lack of SHP-2 had no measurable effect on TCR-CD28-induced IL-2 production (Figure 5C, left panel). When Jurkat-PD-1^OST^ and Jurkat-PD-1^OST^ SHP-2^–^ clones were stimulated with Raji-PD-L1 + SEE, IL-2 production was abolished regardless of the presence or absence of SHP-2. Therefore, PD-1 was still capable of coinhibition in the absence of SHP-2. Comparison of IL-2 production by Jurkat-PD-1^OST^ and Jurkat-PD-1^OST^ SHP-1^–^ clones in response to Raji + SEE showed that the lack of SHP-1 enhanced IL-2 production (Figure 5C, middle panel). In contrast to SHP-2, SHP-1 has thus a unique negative regulatory role irrespective of PD-1-PD-L1 engagement, and its presence reduced by approximately 2-fold the IL-2 produced in response to TCR and CD28 engagement. When Jurkat-PD-1^OST^ SHP-1^–^ clones were stimulated with Raji-PD-L1 + SEE, PD-1 was still capable of mediating coinhibition despite the enhanced IL-2 levels produced in absence of SHP-1. Most important, in response to Raji-PD-L1 + SEE, Jurkat-PD-1^OST^ SHP-1^–^ SHP-2^–^ clones retained the capacity to produce IL-2, in amounts not statistically different from those produced in response to Raji + SEE (Figure 5C, right panel). Therefore, no change in PD-1 coinhibition was observed in absence of either SHP-1 or SHP-2, but PD-1 coinhibition was totally ablated in the absence of both SHP-1 and SHP-2, demonstrating that expression of either PD-1-SHP-1 or PD-1-SHP-2 complexes is sufficient to mediate PD-1 coinhibition in Jurkat T cells as measured by its impact on TCR-CD28-induced IL-2 production. It also suggested that none of the additional proteins identified in the PD-1 signalosome (Figures 1 and 3) contribute to PD-1 coinhibition in absence of SHP-1 and SHP-2.

**Figure 6:**
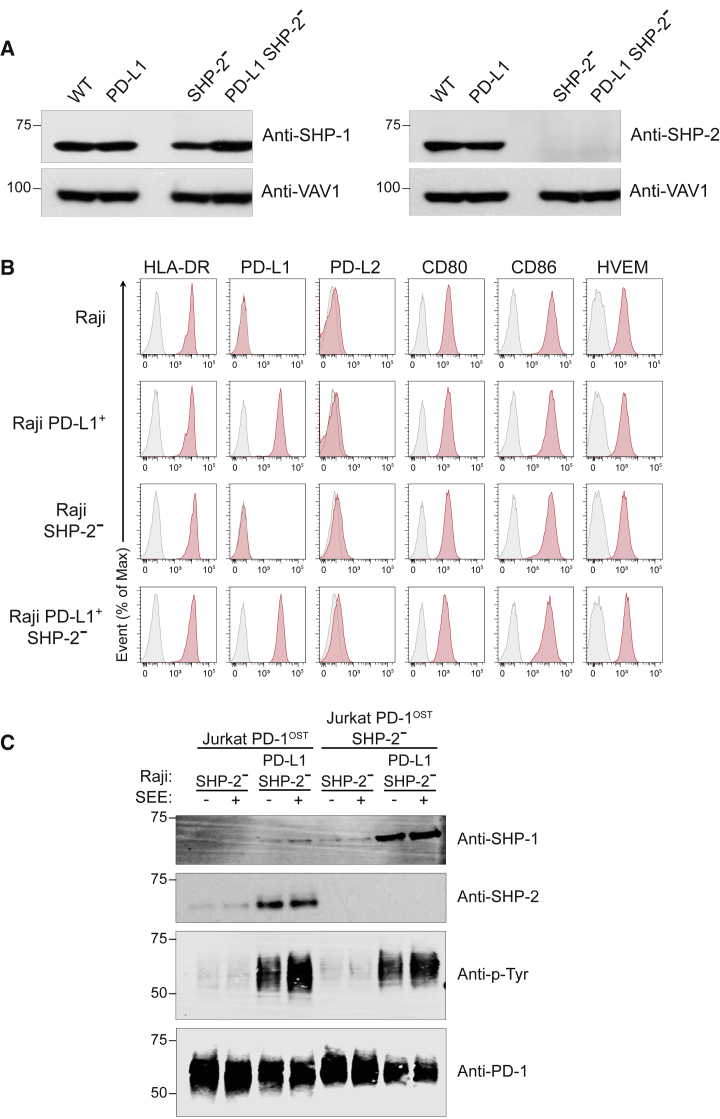
SHP-1 Can Replace SHP-2 for PD-1 Coinhibition in Jurkat T Cells (A) Immunoblot analysis of equal amounts of proteins from total lysates of Raji (WT), Raji PD-L1, Raji SHP-2^–^, and Raji PD-L1 SHP-2^–^ cells probed with antibodies to SHP-1 (right panel), SHP-2 (left panel), or VAV1 (loading control). Left margin, molecular size in kilodaltons. Data are representative of two independent experiments. (B) Raji, Raji PD-L1, Raji SHP-2^–^, and Raji PD-L1 SHP-2^–^ cells were analyzed using flow cytometry for expression of HLA-DR, PD-L1, PD-L2, CD80, CD86, and HVEM. Gray shaded curves correspond to isotype-matched control antibody (negative control), and data are representative of two independent experiments. (C) Jurkat-PD-1^OST^ and Jurkat-PD-1^OST^ SHP-2^–^ cells were stimulated with Raji SHP-2^–^ cells or Raji PD-L1 SHP-2^–^ cells that have been preincubated in the absence (–) or presence (+) of SEE and lysed for 2 min after the initial contact. Immunoblot analysis of equal amounts (90%) of lysates from the specified conditions subjected to affinity purification (AP) on Strep-Tactin-Sepharose beads, followed by elution of proteins with D-biotin, and probed with antibody to anti-SHP-1, anti-SHP-2, and phosphorylated proteins (Anti-p-Tyr). Also shown is immunoblot analysis of equal amounts (10%) of total lysates of the specified cells probed with anti-PD-1 antibody (loading control). Left margin, molecular size in kilodaltons (kDa). Data are representative of two independent experiments.

We determined next whether the unabated PD-1 coinhibition observed in absence of SHP-2 (Figure 5C, left panel) correlated with the presence of increased numbers of PD-1-SHP-1 complexes. Considering that SHP-2 showed a strong propensity to bind to phosphorylated PD-1 molecules over SHP-1 (Hui et al., 2017) and taking into account the dynamic nature of the signalosomes assembling in T cells (Bunnell et al., 2002, Taylor et al., 2017; Figure S4), we specifically used Raji-PD-L1 and control Raji cells rendered SHP-2^–^ via CRISPR-Cas9 editing (Figures 6A and 6B). In response to Raji-PD-L1 SHP-2^–^ + SEE, PD-1-OST molecules expressed in Jurkat-PD-1^OST^ cells primarily interacted with SHP-2 molecules, and only minute amounts of SHP-1 were capable of interacting with them (Figure 6C), a result congruent with our AP-SWATH-MS analysis (Figures 1 and 4). PD-1-SHP-1 complexes were, however, readily detectable in Jurkat-PD-1^OST^ SHP-2^–^ T cells following a 2 min contact with Raji PD-L1 SHP-2^–^ + SEE (Figure 6C). Therefore, in the absence of SHP-2, phosphorylated PD-1 molecules are capable of recruiting increased amounts of SHP-1, allowing them to dampen IL-2 production with a magnitude comparable with the PD-1-SHP-2 complexes that predominantly assemble under physiological conditions (Figure 5C).

### Targets of the PD-1-SHP-2 and PD-1-SHP-1 Complexes at the T Cell-APC Interface

**Figure 7:**
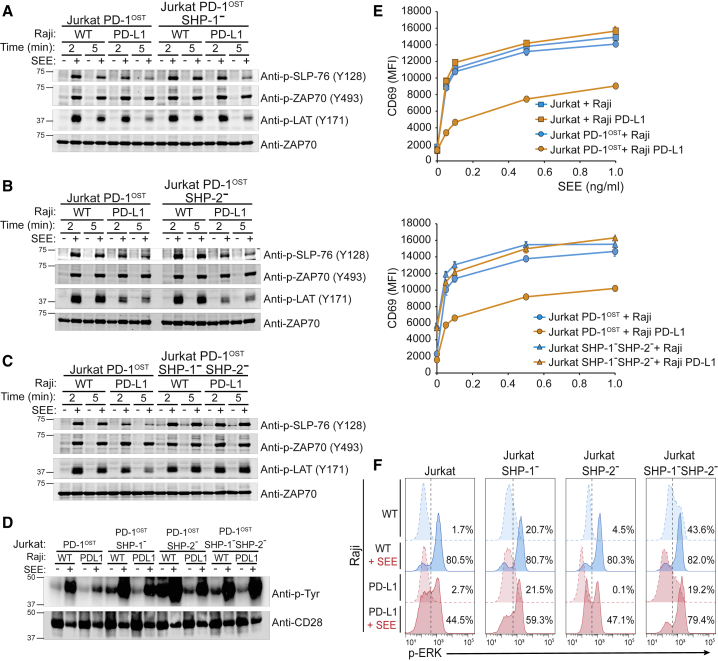
Signaling Pathways Affected by PD-1 Engagement at T Cell-APC Interface in the Presence of SHP-1 and SHP-2, Either SHP-1 or SHP-2, or Neither SHP-1 nor SHP-2 Jurkat-PD-1^OST^, Jurkat-PD-1^OST^ SHP-1^–^, Jurkat-PD-1^OST^ SHP-2^–^, and Jurkat-PD-1^OST^ SHP-1^–^SHP-2^–^ cells were stimulated with Raji cells or Raji-PD-L1 cells that were preincubated in the absence (–) or presence (+) of 200 ng/mL SEE. The various T cell-B cell combinations were lysed after been kept in contact for 2 and 5 min at 37°C. (A) Immunoblot analysis of equal amounts of proteins from total lysates of Jurkat-PD-1^OST^ cells expressing or deprived of SHP-1, probed with phospho-tyrosine-specific antibodies directed against SLP-76 pY128, ZAP-70 pY493 and LAT pY171, and with anti-ZAP-70 antibody (loading control). Data in (A) to (D) are representative of two independent experiments. (B) Immunoblot analysis of equal amounts of proteins from total lysates of Jurkat-PD-1^OST^ cells expressing or deprived of SHP-2, probed as in (A). (C) Immunoblot analysis of equal amounts of proteins from total lysates of Jurkat-PD-1^OST^ cells expressing or deprived of both SHP-1 and SHP-2, probed as in (A). (D) CD28 was immunoprecipitated from equal amounts of proteins from total lysates of the specified Jurkat cells and analyzed in immunoblots probed with antibody to phosphorylated proteins (Anti-p-Tyr) and with anti-CD28 antibody (loading control). (E) Jurkat-PD-1^OST^ and Jurkat-PD-1^OST^ SHP-1^–^SHP-2^–^ cells were stimulated with either Raji cells or Raji-PD-L1 cells that were preincubated in the absence (0) or presence of the specified amounts of SEE. For each condition, CD69 upregulation was measured using fluorescence-activated cell sorting (FACS) 24 h after stimulation. Data are representative of two independent experiments, and mean and SEM are shown. MFI, mean fluorescence intensity. (F) Jurkat-PD-1^OST^, Jurkat-PD-1^OST^ SHP-1^–^, Jurkat-PD-1^OST^ SHP-2^–^, and Jurkat-PD-1^OST^ SHP-1^–^SHP-2^–^ cells were stimulated as in (E), and ERK phosphorylation was measured using FACS 5 min after contact of the specified T and B cells. Numbers indicate the percentage of phospho-ERK^+^ cells. Data are representative of two independent experiments.

The possibility to deconstruct the PD-1 signalosome of Jurkat-PD-1^OST^ cells into PD-1-SHP-2 and PD-1-SHP-1 complexes allowed us to assess whether they dephosphorylated similar substrates when acting at a physiological T cell-APC interface. Representative Jurkat-PD-1^OST^ SHP-1^–^ and Jurkat-PD-1^OST^ SHP-2^–^ clones were selected and stimulated with Raji or Raji-PD-L1 cells plus or minus SEE. Jurkat-PD-1^OST^ and Jurkat-PD-1^OST^ SHP-1^–^ SHP-2^–^ cells were processed in parallel and used as “positive” and “negative” controls, respectively. Comparison of Jurkat-PD-1^OST^ cells stimulated with Raji or Raji-PD-L1 cells + SEE for 5 min showed that PD-1 engagement resulted in reduced levels of tyrosine phosphorylation of CD28 and of the LAT and SLP76 adaptors, whereas no change was detected for ZAP-70 (Figures 7A–7D). Following stimulation for 5 min with Raji-PD-L1 + SEE, Jurkat-PD-1^OST^ SHP-1^–^ and Jurkat-PD-1^OST^ SHP-2^–^ cells showed PD-1-dependent dephosphorylation events similar to those of Jurkat-PD-1^OST^ cells (Figures 7A–7D), a finding consistent with their expression of functional PD-1 molecules (Figure 5C, left and middle panels). In contrast and in line with their lack of functional PD-1 molecules (Figure 5C, right panel), none of the PD-1-dependent dephosphorylation events documented above occurred in Jurkat-PD-1^OST^ SHP-1^–^ SHP-2^–^ cells stimulated with Raji-PD-L1 + SEE (Figures 7A–7D). Therefore, both the PD-1-SHP-2 and PD-1-SHP-1 complexes were capable of targeting the TCR and CD28 signaling pathways when analyzed at a T cell-APC interface.

CD69 is upregulated upon interaction of Jurkat T cells and Raji cells + SEE. It requires TCR engagement and occurs independently of CD28 signaling (Tian et al., 2015). Consistent with our biochemical data suggesting that the TCR signaling pathway itself is a target for PD-1-coinhibition (Figures 7A–7D), upregulation of CD69 was inhibited when Jurkat-PD-1^OST^ cells were stimulated by Raji-PD-L1 cells + SEE (Figure 7E). As expected, CD69 induction was regained when Jurkat-PD-1^OST^ SHP-1^–^ SHP-2^–^ cells were stimulated with Raji-PD-L1 cells + SEE (Figure 7E). CD69 expression requires TCR-mediated activation of the Ras-MAK-ERK signaling pathway (Tian et al., 2015). Accordingly, co-engagement of the TCR and PD-1 pathways diminished the frequency of Jurkat T cells expressing phosphorylated ERK molecules compared with engagement of the TCR alone, and this reduction required the expression of SHP-1 or SHP-2 (Figure 7F). In support of the view that SHP-1 exerts PD-1 independent negative effects on T cell activation (Figure 5C), baseline levels of phosphorylated ERK molecules were increased in Jurkat-PD-1^OST^ SHP-1^–^ cells and Jurkat-PD-1^OST^ SHP-1^–^ SHP-2^–^ cells (Figure 7F). In conclusion, using physiological T cell-APC interactions, we established that several components of the TCR signaling pathway as well as CD28 itself were sensitive to dephosphorylation by PD-1, irrespective of its use of SHP-1 or SHP-2.

## Discussion

Using quantitative interactomics, we defined the composition, stoichiometry, and dynamics of the PD-1 and BTLA coinhibitory signalosomes in primary CD4^+^ T cells. In the case of PD-1, we extended this analysis to PD-1^+^ Jurkat T cells activated upon contact with superantigen-laden PD-L1^+^ APC. The possibility to determine by AP-MS the composition of the PD-1 interactome at a physiological T cell-APC interface goes beyond two recent studies that used a synthetic PD-1 cytoplasmic tail to affinity purify interacting proteins from lysates of Jurkat T cells (Meng et al., 2018, Peled et al., 2018). We showed that under physiological conditions, PD-1 coinhibition relied almost exclusively on the use of SHP-2. However, under experimental conditions resulting in SHP-2 deficiency, SHP-1 was capable of replacing SHP-2 and mediating PD-1 coinhibition, thereby explaining the paradoxical finding that SHP-2 is dispensable for PD-1 signaling *in vivo* (Rota et al., 2018). A highly potent small-molecule inhibitor that stabilizes SHP-2 in its auto-inhibited conformation has been recently developed for treatment of cancers and suggested to be capable of blocking PD-1 coinhibition (Chen et al., 2016). Our data, demonstrating that SHP-1 is capable of mediating PD-1 coinhibition in absence of SHP-2, suggest, however, that upon treatment with this SHP-2-selective inhibitor, SHP-1 will be able to take over and PD-1 coinhibition will remain unabated.

Our finding that SHP-2 molecules outcompete SHP-1 molecules for binding to phosphorylated PD-1 molecules under physiological conditions is consistent with a fluorescence resonance energy transfer (FRET)-based analysis conducted in a cell-free reconstitution system and demonstrating that phosphorylated PD-1 shows 29-fold binding selectivity toward SHP-2 over SHP-1 (Hui et al., 2017). The propensity of phosphorylated PD-1 molecules to bind SHP-2 over SHP-1 is even more striking considering that SHP-1 is 12-fold more abundant than SHP-2 in mouse effector T cells (Hukelmann et al., 2016), a ratio that holds true in ten distinct human T cell populations analyzed in steady and activated states (Rieckmann et al., 2017). However, the fact that SHP-1 is more abundant than SHP-2 in T cells must be tempered by the observation that SHP-1 adopts a tighter auto-inhibited conformation than SHP-2, which likely decreases its actual concentration capable of binding to PD-1 (Goyette et al., 2017, Hui et al., 2017). Interestingly, BTLA uses SHP-1 and SHP-2 in a more balanced manner than PD-1. This difference in SHP-1 and SHP-2 use likely reflects subtle structural differences intrinsic to the PD-1 and BTLA cytosolic tail. In T cells, SHP-1 is thought to act as a direct negative regulator of the TCR signal transduction network via its recruitment by ZAP-70, LCK, or THEMIS1 (Choi et al., 2010, Paster et al., 2015, Plas et al., 1996, Stefanová et al., 2003). Consistent with this negative regulatory role, in the absence of PD-1-PDL1 engagement, TCR-CD28-triggered IL-2 production was increased 2-fold by the lack of SHP-1. Likewise, baseline levels of phosphorylated ERK molecules were increased in absence of SHP-1. Therefore, in contrast to SHP-2, SHP-1 can, in addition to its contribution to PD-1 coinhibition, negatively regulate TCR signals in a manner independent of coinhibitory receptor signaling.

Maximal PD-1 phosphorylation by LCK molecules and subsequent SHP-2 recruitment required TCR and PD-1 co-engagement (Hui et al., 2017, Yokosuka et al., 2012). PD-1 phosphorylation and SHP-2 recruitment also occurred, but with a lower magnitude, following PD-1-PD-L1 engagement alone. This is consistent with a recent study demonstrating that upon T cell-APC encounter, the PD-1-PD-L1 clusters can assemble in the absence of TCR engagement and by excluding the CD45 PTPase from the contact zone shift the local kinase-phosphatase balance in favor of PD-1 phosphorylation (Carbone et al., 2017). Therefore, it is likely that PD-1 not only initiates a negative feedback loop in response to TCR signals but also contributes through its sole engagement to increase the threshold that needs to be overcome by TCR signals to trigger T cell responses. Such possibility might particularly afflict exhausted T cells because of their expression of 3- to 10-fold more PD-1 than acute effector T cells (Wei et al., 2013) and maintain them in a state of reduced reactivity toward exogenous antigens.

Although a recent study suggests that the CD28 signaling pathway is the primary target of PD-1 coinhibition (Hui et al., 2017), our biochemical and functional analyses showed that the PD-1-SHP-2 and PD-1-SHP-1 complexes target both the TCR and CD28 signaling pathways, a finding consistent with former studies showing that the TCR signaling pathway can also be subjected to PD-1 coinhibition (Sheppard et al., 2004, Wei et al., 2013, Yokosuka et al., 2012) and with recent data demonstrating that PD-1 inhibited TCR-induced cytokine production in absence CD28 costimulation (Mizuno et al., 2019). The PD-1 ability to inhibit the initiation of both the TCR and CD28 signaling pathways explains the pleiotropic functional outcomes that result from its engagement (Sharpe and Pauken, 2018) and accounts for the ability of PD-1 to suppress TILs directed against non-hematopoietic tumor cells that express ligands of PD-1 but not those of CD28.

The overlap noted in the substrates targeted by PD-1-SHP-1 and PD-1-SHP-2 suggests that the contrasted usage of SHP-1 and SHP-2 by PD-1 and BTLA might be of limited functional consequence. Accordingly, the TCR and CD28 signaling pathways of TILs coexpressing PD-1 and BTLA (Baitsch et al., 2012) can be concomitantly subjected to two layers of redundant coinhibitory mechanisms. As a result, engagement of the BTLA pathway is likely to mask the phenotypic consequences of blocking the PD-1 pathway, and coadministration of PD-1 and BTLA antibodies to patients should augment the therapeutic benefit of PD-1 blockade, as illustrated by *in vivo* studies in the mouse (Ahrends et al., 2017) and *in vitro* studies in humans (Stecher et al., 2017). Provided further confirmation of their palmitoylation status, a fraction of BTLA molecules might, however, bring SHP-1 and SHP-2 in contiguity with substrates that are uniquely present in lipid microdomains and that are out of reach for PD-1. Note that conditions in which PD-1 and BTLA ligands show non-overlapping expression make the existence of the PD-1 and BTLA paralogs necessary. In cancer immunotherapy, combining antibodies to PD-1 and BTLA might thus also be of therapeutic benefit in conditions in which a tumor mass is heterogeneous in term of PD-1 and BTLA ligand expression.

Two recent AP-MS studies aimed at uncovering proteins associating with PD-1 used lysates of Jurkat T cells and a recombinant bait made of a synthetic version of the PD-1 cytoplasmic tail. In one study, SAP (SH2D1A), a protein that interacts with SLAM family receptors, was found associated with tyrosine-phosphorylated PD-1 cytoplasmic tail in one of three biological replicates (Peled et al., 2018), whereas in the other study the FBXO38 E3 ligase associated with PD-1 baits of undefined tyrosine phosphorylation status (Meng et al., 2018). We failed to identify both SAP and FBXO38 using PD-1 molecules expressed at physiological levels in intact T cells, and the lack of statistical and stoichiometric information in the two studies relying on synthetic PD-1 cytoplasmic tail and Jurkat cell lysates makes a comparison with our study difficult.

The two models we used to determine the composition of the PD-1 coinhibitory signalosome rely on mouse primary CD4^+^ T cells that were briefly expanded and stimulated by pervanadate and on human Jurkat T cells that were activated by superantigen-laden PD-L1^+^ APC. Although they provided a convergent picture on the mechanism of PD-1 coinhibition, each has unique drawbacks. Pervanadate-based T cell activation is admittedly a less physiological condition than that relying on T cell-APC contact. However, it unexpectedly resulted in the assembly of a PD-1 signalosome that was similar to that resulting from physiological T-APC contacts in terms of its composition and kinetics of assembly-disassembly with SHP-1, SHP-2, and GRB2. Along that line, a previous AP-MS study of the interactomes assembling around the SLP-76 adaptor following stimulation with anti-CD3 and anti-CD4 antibodies or with pervanadate found 80% overlap in their protein composition (Roncagalli et al., 2014). The convergent view provided by our two models might not reflect the PD-1 signalosome found in T cells of varying differentiation states. For instance, CD8^+^ TILs acquire a state of exhaustion analogous to that elicited by chronic viral infection. Among them, a small cell subset expressing PD-1 and the transcription factor TCF1 (encoded by *Tcf7*) can respond to anti-PD-1 immunotherapy and differentiate into highly cytotoxic, terminally exhausted TILs that mediate long-term tumor control (Im et al., 2016, Miller et al., 2019, Siddiqui et al., 2019). Our present AP-MS pipeline is, however, quite demanding in terms of T cell numbers, and its application to the PD-1^+^TCF1^+^ subpopulation of CD8^+^ TILs that respond to anti-PD-1 treatment will require hundreds of tumor-bearing PD-1-OST mice to isolate such TILs in sufficient number. Therefore, the CRISPR-Cas9-editable, Jurkat-PD-1-Raji-PD-L1 platform constitutes the most tractable model yet to document via AP-MS and at the T-APC interface how PD-1 coinhibition affects TCR-CD28 signals and to disentangle the respective function of PD-1-SHP-2 and PD-1-SHP-1 complexes.

In conclusion, our study illustrates the importance of distinguishing protein-protein interactions that occur using endogenous baits expressed at physiological levels and that do not disrupt the subtle stoichiometry of intracellular signaling complexes (Hein et al., 2015) from those that are possible experimentally in conditions of overexpression or of disrupted cellular architecture. It also shows that by permitting pairwise comparison of coinhibitory signalosomes in primary T cells, quantitative interactomics unveils whether they elicit redundant inhibitory signals and helps decide if a given pair of coinhibitory receptors should be preferred over another during the design of combinations of immunotherapeutic agents.

## STAR★Methods

### Key Resources Table

**Table:** 

REAGENT or RESOURCE	SOURCE	IDENTIFIER
**Antibodies**		

Mouse monoclonal anti-SH-PTP1 (D-11)	Santa Cruz biotechnology	Cat# sc-7289; RRID: AB_628251
Mouse monoclonal anti-SHP2	Cell signaling technology	Cat# 3752; RRID: AB_2300607
Rabbit polyclonal anti-VAV1	Cell signaling technology	Cat# 2502; RRID: AB_2213556
Mouse monoclonam anti-pSLP76 (Y128)	BD Pharmigen	Cat# 558367; RRID: AB_647331
Rabbit polyclonal anti-pZAP70 (Y493)	Cell signaling technology	Cat# 2704; RRID: AB_2217457
Rabbit monoclonal anti-ZAP70	Cell signaling technology	Cat# 2705; RRID: AB_2273231
Rabbit polyclonal anti-pLAT (Y171)	Cell signaling technology	Cat# 3581S; RRID: AB_2157730
Rabbit polyclonal anti-SLP76	Cell signaling technology	Cat# 4958; RRID: AB_2136713
Goat polyclonal anti-CD28	Santa Cruz biotechnology	Cat# sc-1623; RRID: AB_2073867
Mouse anti-human CD28 (CD28.2)	Biolegend	Cat# 302902; RRID: AB_314304
Mouse monoclonal anti-Phosphotyrosine (clone 4G10)	Millipore	Cat# 05-321; RRID: AB_309678
Rabbit monoclonal anti-human PD-1 (D4W2J)	Cell signaling technology	Cat# 86163; RRID: AB_2728833)
Rat polyclonal anti-mouse PD-1 (RMP1-14)	Biolegend	Cat# 114102; RRID: AB_313573
Goat polyclonal anti-mouse BTLA	R&D systems	Cat# AF3007; RRID: AB_2243788
Goat anti-rabbit IgG CF770	Biotium	Cat# 20078; RRID: AB_10563034
Goat antimouse IgG CF680	Biotium	Cat# 20065; RRID: AB_10557108
Sheet anti-mouse HRP	GE Healthcare	Cat# NA9310-1ml; RRID: AB_772193
Donkey anti-rabbit HRP	GE Healthcare	Cat# NA9340-1ml; RRID: AB_772191
Purified anti-CD28 (37-51)	Exbio Praha	Cat# 12-597-C500; RRID: AB_10734810
Purified anti-CD3 (145-2C11)	Exbio Praha	Cat# 12-578-C500; RRID: AB_10738256
Rabbit monoclonal anti-p44/42 MAPK (197G2) PE	Cell signaling technology	Cat# 14095; RRID: AB_2728834
anti-mouse CD19 (6D5) APC Fire750	Biolegend	Cat# 115558; RRID: AB_2572120
anti-mouse CD25 (PC61) FITC	Biolegend	Cat# 102006; RRID: AB_312855
anti-mouse CD3e (145-2C11) PE	Biolegend	Cat# 100307; RRID: AB_312672
anti-mouse CD4 (RMA4-5) BV650	BD Biosciences	Cat# 563747; RRID: AB_2716859
anti-mouse CD44 (IM7) PE-Cy7	BD Biosciences	Cat# 560569; RRID: AB_1727484
anti-mouse CD5 (53-7.3) Pe-Cy5	BD Biosciences	Cat# 553024; RRID: AB_394562
anti-mouse CD62L (MEL-14) FITC	BD Biosciences	Cat# 553150; RRID: AB_394665
anti-mouse CD8a (53-6.7) AF700	Biolegend	Cat# 100730; RRID: AB 493703
anti-mouse CD272 (BTLA, 8F4) PE	Biolegend	Cat# 134804; RRID: AB_1731884
anti-mouse CD279 (PD-1,RPM1-30) PE	Biolegend	Cat# 109104; RRID: AB_313421
anti-mouse IgG1 κ isotype control (P3.6.2.8.1) PE	eBioscience	Cat# 12-4714-82; RRID: AB_470060
Anti-rat IgG2b κ isotype control PE	BD Biosciences	Cat# 553989; RRID: AB_10049479
anti-mouse TCRβ (H57-597) BV421	BD Biosciences	Cat# 562839; RRID: AB_2737830
anti-human CD69 (FN50) PE	Biolegend	Cat# 310906; RRID: AB_314841
anti-human CD28 (CD28.2) PE	BD Biosciences	Cat# 555729; RRID: AB_396072
anti-human CD3ε (OKT3) APC	Invitrogen	Cat# 17-0037-42; RRID: AB_1907372
anti-human CD5 (UCHT2) PE-Cy7	Biolegend	Cat# 300622; RRID: AB_2275812
anti-human CD5 (UCHT2) APC	Biolegend	Cat# 300612; RRID: AB_314098
anti-human CD19 (HIB19) FITC	BD Biosciences	Cat# 555412; RRID: AB_395812
anti-human CD19 (HIB19) BV421	BD Biosciences	Cat# 562441; RRID: AB_11154587
anti-human CD86 (IT2.2) APC	Biolegend	Cat# 305411; RRID: AB_493232
anti-human CD80 (2D10) BV421	Biolegend	Cat# 305221; RRID: AB_10899567
anti-human CD272 (BTLA, MIH26) PE-Cy7	Biolegend	Cat# 344515; RRID: AB_2629565
anti-human CD270 (HVEM, TR2) PE-Cy7	Biolegend	Cat# 318809; RRID: AB_2565254
anti-human CD279 (PD-1, EH12.2H7) BV421	Biolegend	Cat# 329920; RRID: AB_10960742
anti-human CD274 (PD-L1, MIH1) eFluor450	eBioscience	Cat# 48-5983-42; RRID: AB_2574091
anti-human CD273 (PD-L2, MIH18) PE	Biolegend	Cat# 345505; RRID: AB_1953231
anti-human HLA-DR (LN3) PE	eBioscience	Cat# 12-9956-42; RRID: AB_10698015

**Chemicals, Peptides, and Recombinant Proteins**		

Sodium Orthovanadate	Acros organics	Cat# 205330500
Hydrogen Peroxide Solution	Sigma Aldrich	Cat# 216763
Phorbol 12-myristate 13-acetate (PMA)	EMD Millipore	Cat# 19-144
Ionomycin calcium salt from Streptomyces conglobatus	Sigma Aldrich	Cat# I0634
Strep-Tactin Sepharose beads	IBA Lifesciences	Cat# 2-1201-010
D-biotin	Sigma Aldrich	Cat# B4501
PNGaseF	New England Biolabs	Cat# P0704S
n-Dodecyl-β-D-maltoside	Merck	Cat# 324355
Aprotinin	Roche	Cat# 10981532001
Leupeptin	Roche	Cat# 11034626001
PMSF	Roche	Cat# 2088311
Nα-Tosyl-L-lysine chloromethyl ketone hydrochloride	Sigma-Aldrich	Cat# T7254
N-p-Tosyl-L-phenylalanine chloromethyl ketone	Sigma-Aldrich	Cat# T4376
Enterotoxin type E Recombinant Protein - 1 mg (E-Coli)	Mybiosource	Cat# mbs1112600
iRT peptides	Biognosys	N/A
Trypsin	Promega	Cat#V5113

**Critical Commercial Assays**		

DB UNTOUCHED MOUSE CD4 CELLS KIT	Life technologies	Cat# 11415D
CellTiter-Glo® Luminescent Cell Viability Assay	Promega	Cat# G7571
Cell line nucleofector kit V	Lonza	Cat# VCA-1003
Human IL-2 DuoSet ELISA	R&D Systems	Cat# DY202

**Experimental Models: Cell Lines**		

Jurkat, Clone E6-1	ATCC	Cat# TIB-152; RRID: CVCL_0367
Jurkat-PD-1	This paper	N/A
Jurkat-PD-1^OST^	This paper	N/A
Jurkat-PD-1^OST^ SHP-1^–^	This paper	N/A
Jurkat-PD-1^OST^ SHP-2^–^	This paper	N/A
Jurkat-PD-1^OST^ SHP-1^–^ SHP-2^–^	This paper	N/A
Raji	ATCC	Cat# CCL-86; RRID:CVCL_0511
Raji-PD-L1	This paper	N/A
Raji-PD-L1 SHP-2^–^	This paper	N/A

**Experimental Models: Organisms/Strains**		

PD-1^OST^ mice	This paper	B6-*Pdcd1*^*tm1Ciphe*^
BTLA^OST^ mice	This paper	B6-*Btla*^*tm1Ciphe*^

**Oligonucleotides**		

PDL1 Fw CGGAATTCCGGCCACCATGAGGATAT TTGCTGTC	This paper	N/A
PDL1 Rev GCTTTGTTTAAACGGCGAATGCGGCC GCTA TTACGTCTCCTCCAAATGTGTATCACTTTGC	This paper	N/A

**Recombinant DNA**		

pEF6/Myc-His	Thermofisher Invitrogen	Cat# V96220

**Software and Algorithms**		

R V3.3	GNU General public license	https://www.r-project.org/
R studio V1.0.136	GNU General public license	https://www.rstudio.com/
FlowJo V10	TreeStar, FlowJo LLC, Ashland, Oregon	https://www.flowjo.com/
GraphPad Prism 7	GraphPad Software, Inc., California	https://www.graphpad.com/scientific-software/prism/
FACSDiva software v8	BD FACSDivaTM	http://www.bdbiosciences.com/us/instruments/research/software/
Spectronaut X	Biognosys, Schlieren, Switzerland	https://biognosys.com
MaxQuant v.1.5.2.8	Max Plank Institute of Biochemistry, Germany	https://www.maxquant.org/
mapDIA	PMID: 26381204	https://sourceforge.net/projects/mapdia/
PECA package	PMID: 28724900	http://bioconductor.org/packages/PECA/

### Contact for Reagent and Resource Sharing

Further information and requests for resources and reagents should be directed to and will be fulfilled by the Lead Contact, Bernard Malissen (bernardm@ciml.univ-mrs.fr).

### Experimental Models and Subject Details

#### Mice

Mice were maintained in specific pathogen-free conditions and used at 8 to12 week of age. Both sexes were used in all the reported experiments. Generation of the PD-1^OST^ (B6-*Pdcd1*^tm3Ciphe^) and BTLA^OST^ (B6-*Btla*^Tm2Ciphe^) mice is described below.

#### Animal experimental guidelines

Mice were handled in accordance with national and European laws for laboratory animal welfare and experimentation (EEC Council Directive 2010/63/EU, September 2010), and protocols approved by the Marseille Ethical Committee for Animal Experimentation.

#### Human cell lines

The Jurkat human leukemic T cell line and the Raji lymphoblastoid B cell line were provided by A. Weiss (University of California San Francisco, CA) and originated from American Type Culture Collection. Jurkat and Raji cells were maintained in DMEM medium supplemented with 10% fetal bovine serum, 100 U/ml of Penicilin and 100 μg/mL Streptomycin.

### Method Details

#### Pdcd1^OST^ and Btla^OST^ targeting vectors

A double-stranded DNA repair template (targeting vector) with 5′ and 3′ homology arms of 885 bp and 1000 bp, respectively, was assembled to edit the *Pdcd1* gene that codes for PD-1 molecules. It included a Twin-Strep-tag-coding sequence (Junttila et al., 2005) inserted at the end of the last exon (exon 5) of the *Pdcd1* gene and a self-excising ACN cassette (Roncagalli et al., 2014) that was introduced at the beginning of the 3′ UTR sequence. The final targeting vector was abutted to a cassette coding for the diphtheria toxin fragment A (Soriano, 1997). A similar strategy was used for preparing a *Btla*^OST^ targeting vector. The protospacer adjacent motif (PAM) present in each targeting vector was destroyed via a silent mutation to prevent CRISPR-Cas9 cleavage.

#### Isolation of recombinant embryonic stem (ES) cell clones

sgRNA-specifying oligonucleotide sequences (*Pdcd1*^OST^ ES cells: 5′-CACCGCTGAAGAATCTGGTCAAAG-3′ and 5′-AAACCTTTGACCAGATTCTTCAGC-3′ and *Btla*^OST^ ES cells: 5′-CACCGTTAAACCTGCCACTGAGCC-3′ and 5′- AAACGGCTCAGTGGCAGGTTTAAC-3′) were chosen to minimize the likelihood of off-target sequence using publicly available tool (http://crispor.tefor.net). Upon annealing, each pair of sgRNA-specifying oligonucleotides generated overhangs for ligation into the BbsI site of plasmid pX330 (pSpCas9; Addgene, plasmid ID 42230). JM8.F6 C57BL/6N ES cells (Pettitt et al., 2009) were electroporated with 20 μg of *Pdcd1*^OST^ or *Btla*^OST^ targeting vector and 2.5 μg of the matched pX330-sgRNA plasmid. After selection in G418, ES cell clones were screened for proper homologous recombination by Southern blot or PCR analysis. A *neo*^r^ specific probe was used to ensure that adventitious non-homologous recombination events had not occurred in the selected ES clones.

#### Production of knock-in mice

Mutant ES cells were injected into BalbC/N blastocysts. In the case of the *Pdcd1*^OST^ allele, following germ-line transmission, screening for proper deletion of the ACN cassette and for the presence of the sequence coding for the OST was performed by PCR using the following pair of primers: sense 5′-CATTGTCTTCACTGAAGGGC-3′ and antisense 5′-ATGTGCTGGAATTGGTGCAG-3′. This pair of primers amplified a 456 bp band and a 275 bp band in the case of the *Pdcd1*^OST^ allele and of the wild-type *Pdcd1* allele, respectively. In the case of the *Btla*^OST^ allele, the following pair of PCR primers was used: sense 5′-TTGAACCATTGTGTTATTGG-3′ and antisense 5′-GCACAGAGCATCTTAATTGAAA-3′. This pair of primers amplified a 389 bp band and a 208 bp band in the case of the *Btla*^OST^ allele and of the wild-type *Btla* allele, respectively.

#### Flow cytometry of mouse T and B cells

Stained cells from spleen and lymph nodes were analyzed using an LSRII system (BD Biosciences), and data were analyzed with a Diva software (BD Biosciences). Cell viability was evaluated using SYTOX Blue (Life Technologies). The list of antibodies used for flow cytometry of mouse T and B cells can be found in the Key Resource Table.

#### Mouse CD4^+^ T cell proliferation

Purified CD4^+^ T cells were stimulated with plate-bound anti-CD3 (145-2C11; Exbio) and soluble anti-CD28 (37-51; Exbio) antibodies. After 48 h of culture, T cell proliferation was assessed with CellTiter-Glo® Luminescent (Promega). The resulting luminescence, which is proportional to the ATP content of the culture, was measured with a Victor 2 luminometer (Wallac, Perkin Elmer Life Science).

#### Mouse CD4^+^ T cell isolation and short-term expansion prior to AP-MS analysis

CD4^+^ T cells were purified from pooled lymph nodes and spleens with Dynabeads Untouched Mouse CD4^+^ T Cell Kits (Life Technologies); cell purity was > 95%. CD4^+^ T cells purified from PD-1^OST^ mice were expanded with plate-bound anti-CD3 (145-2C11, 5 μg/ml) and anti-CD28 (37-51; 1 μg/ml) antibodies both from Exbio Praha. Cultures were supplemented with IL-2 (5–10 U/ml) 48 h after their initiation, CD4^+^ T cells were harvested 72h after culture initiation, splitted and grown overnight in the presence of IL-2 alone prior to AP-MS analysis. Wild-type CD4^+^ T cells were subjected to the same expansion protocol and used as controls in AP-MS experiments. CD4^+^ T cells purified from BTLA^OST^ mice were expanded with plate-bound anti-CD3 (145-2C11, 5 μg/ml) and soluble anti-CD28 (37-51; 1 μg/ml) antibodies. CD4^+^ T cells were harvested, 48 h after culture initiation, splitted and grown for 24 h in the presence of IL-2 alone prior to AP-MS analysis. Wild-type CD4^+^ T cells were subjected to the same expansion protocol and used as controls in AP-MS experiments.

#### Stimulation and lysis of short-term expanded mouse CD4^+^ T cells prior to AP-MS analysis

Short-term expanded CD4^+^ T cells (100 × 10^6^) from either PD-1^OST^ or BTLA^OST^ mice and from wild-type mice were kept at 37°C for 5 min and either left unstimulated or stimulated with pervanadate (Roncagalli et al., 2014) for the specified times. Stimulation was stopped by the addition of a twice-concentrated lysis buffer (100 mM Tris, pH 7.5, 270 mM NaCl, 1 mM EDTA, 20% glycerol, 0.4% n-dodecyl-β-maltoside) supplemented with protease and phosphatase inhibitors. After 10 min of incubation on ice, cell lysates were centrifuged at 14,000 rpm for 5 min at 4°C. Postnuclear lysates were then used for affinity purification as described below.

#### Sensitivity of PD-1 and BTLA molecule to glycopeptidase F

To determine the sensitivity of PD-1 and BTLA molecule to glycopeptidase F (PNGase F; EC 3.2.2.18), a glycoaminidase that cleaves the link between asparagine and N-acetylglucosamine, PD-1-OST and BTLA-OST molecules were affinity purified from CD4^+^ T cells from PD-1^OST^ and BTLA^OST^ mice using Sepharose beads coupled to Strep-Tactin. They were then treated with 1 unit of PNGase F (New England Biolabs), reduced, and analyzed on an SDS polyacrylamide gel.

#### Transfection of Jurkat and Raji cells

A synthetic cDNA coding for wild-type human PD-1 molecules (Uniprot Q15116) with or without a Twin-Strep-tag at their carboxyl-terminus was cloned into vector pEF6/MyC-HisA (Thermo Fisher Scientific; Cat. n° V96220). Jurkat cells were transfected with an Amaxa apparatus, and sorted to express homogeneous levels of PD-1 at their surface. A synthetic cDNA coding for wild-type human PD-L1 molecules (Uniprot Q9NZQ7) was cloned into vector pEF6/MyC-HisA. Raji B cells were transfected with an Amaxa apparatus, and sorted to express homogeneous levels of PD-L1 at their surface.

#### Flow cytometry of Jurkat and Raji cells

The list of antibodies used to analyze Jurkat and Raji cells by flow cytometry can be found in the Key Resource Table.

#### Small-scale Jurkat T cell stimulation

Jurkat T cells (10^5^) were stimulated by co-culture with Raji cells (0.5 × 10^5^) presenting the superantigen SEE (Toxin Technology), which binds to both the TCR and MHC class II molecules. IL-2 production was measured by ELISA (R&D Systems), whereas CD69 induction and ERK1/2 phosphorylation were measured by flow cytometry.

#### Large-scale Jurkat T cell stimulation for AP-MS analysis

Raji B cells (or their transfected or edited variants) were incubated at 37°C for 30 min with 200 ng/mL SEE, washed and resuspended at a concentration of 200 × 10^6^/mL. Jurkat T cells (or their transfected or edited variants) were distributed into microcentrifuge tubes (20 × 10^6^ in a volume of 0.1 ml). Jurkat T cells and Raji B cells were then kept at 37°C for 10 min. 0.1 mL of Raji cell suspension was added to the microcentrifuge tubes and rapidly mixed together with Jurkat T cells. To promote cell-cell contact, microcentrifuge tubes were immediately centrifuged at 400 g for 30 s at room temperature, and then kept at 37°C for the specified time without resuspending the cell pellet. Under those conditions, analysis of ERK phosphorylation by flow cytometry showed that up to 80% of Jurkat T cells were activated via their TCR (Figure 7F). The cell pellet was lysed by addition of 0.2 mL of twice-concentrated lysis buffer (100 mM Tris, pH 7.5, 270 mM NaCl, 1 mM EDTA, 20% glycerol, 0.4% n-dodecyl-β-maltoside) supplemented with protease (Sigma) and phosphatase (Roche) inhibitors and processed for AP-MS as described below.

#### SILAC labeling of Raji cells

For SILAC labeling, the RajiSHP2- and Raji PDL1+ SHP2- cells were cultured in arginine- and lysine-free RPMI 1640 media (Thermoscientific) supplied with 10% (vol/vol) dialyzed FBS (Thermoscientific), and L-[^13^C_6_, ^15^N_2_]-lysine, and L-[^13^C_6_, ^15^N_4_]-arginine (Thermoscientific). Prior to be used for Jurkat T cell stimulation, the Raji cells were grown for at least eight passages to ensure full isotope incorporation.

#### Stimulation of Jurkat T cells with SILAC-labeled Raji cells and sample preparation for AP-MS

Large scale stimulation of Jurkat T cell with SILAC-labeled Raji cells and sample preparation for AP-MS were conducted as described above for experiments involving unlabeled Raji cells.

#### Biochemical analysis of Jurkat T cells

Immunoblot analysis was performed as described (Roncagalli et al., 2014). The list of antibodies used for biochemical analysis of Jurkat cells can be found in the Key Resource Table.

#### CRISPR-Cas9-based genome editing of Jurkat T and Raji B cells

We developed a fast-track approach that permits to introduce a single or multiple biallelic null mutations in large numbers of independent clones of Jurkat T cells. Briefly, sgRNA-specifying oligonucleotide sequences were chosen to minimize the likelihood of off-target cleavage based on publicly available on-line tools (https://www.dna20.com/products). The following pairs of sgRNA-specifying oligonucleotide sequences have been used: *Ptpn6*^–^: 5′- cacc*g*acctgatcccccaccctgc −3′ and 5′- aaacgcagggtgggggatcaggt*c* −3′ (it targets exon 3 that is shared between the different *Ptpn6* transcripts); *Ptpn11*^–^: 5′- cacc*g*gtttcatggacatctctct −3′ and 5′- aaacagagagatgtccatgaaac*c* −3′ (it targets exon 4 that is shared between the different *Ptpn11* transcripts). The oligonucleotide guides contained cacc and aacc overhangs for cloning into *BbsI* sites of plasmid pX330 (pSpCas9; Addgene plasmid ID 42230) or pX458 (pSpCas9(BB)-2A-GFP; Addgene plasmid ID 48138), and a G-C base pair (italics) was added at the 5′ end of the guide sequence for T7 transcription. To obtain in a single round of electroporation large numbers of independent Jurkat T cell clones with bi-allelic inactivation of *Ptpn6* or *Ptpn11*, we devised a gene trapping strategy in which a neomycin resistance gene (NeoR)-containing cassette - called ‘bi-allelic KO type 1 cassette’ - is inserted in frame in exon 3 (*Ptpn6*) or 4 (*Ptpn11*) to lead to an early interruption of their open reading frame. Owing to the high cutting efficiency of CRISPR-Cas9, most of the Jurkat T cells that grow following selection in the presence of 2 mg/ml neomycin had the intended bi-allelic inactivation. The bi-allelic KO type 1 cassette codes for a BamHI restriction site, a Gly-Ser-Gly-flanked ‘self cleaving’ P2A peptide, a Met (start) codon, a translatable *loxP*511 sequence, a neomycine resistance gene, a stop codon, a *loxP*511 sequence, a synthetic intron, a polyA sequence and a NotI restriction site. Double-stranded DNA repair templates (‘targeting vector’) containing the ‘bi-allelic KO type 1 cassette’ as well as additional homologous sequence immediately upstream and downstream (‘homology arms’) of the exonic sequence to be targeted were assembled. The size of the left and right homology arms of the targeting vector used for *Ptpn6* and *Ptpn11* inactivation was 500 bp. A silent mutation was introduced in each targeting vector to destroy the corresponding PAM sequence. Jurkat T cells were nucleofected using the Cell Line Nucleofector® Kit V program I-010 for Nucleofector II with 2.5 μg of linearized targeting vector, and 7 μg of pX330-sgRNA or pX335-sgRNA plasmid. Cells were allowed to recover for 48 h and subjected to G418 selection (2 mg/ml). After 72 h of selection, cells were cloned by limiting dilution and each resulting clone screened for proper gene editing using PCR and genomic DNA sequencing. Importantly, the *loxP*511-flanked NeoR cassette used in the first round of edition can be readily deleted via electroporation of StemMACS Cre Recombinase mRNA (Miltenyi Biotec), allowing to proceed to iterative cycles of biallelic edition using NeoR cassettes flanked by *loxP* site variants. Using this fast-track iterative edition process, Jurkat T cells deprived of PTPN11 were derived via a first round of edition and subsequently used to derive Jurkat T cells deprived of both PTPN6 and PTPN11. Raji B cells deprived of PTPN11 were derived as described for Jurkat T cells.

#### Affinity purification of protein complexes

Equal amount of post-nuclear lysates were incubated with prewashed Strep-Tactin Sepharose beads (IBA GmbH) for 1.5 h at 4°C on a rotary wheel. Beads were then washed 5 times with 1 mL of lysis buffer in the absence of detergent and of protease and phosphatase inhibitors. Proteins were eluted from the Strep-Tactin Sepharose beads with 2.5 mM D-biotin, a competitive ligand that binds to Strep-Tactin with a higher affinity than the OST sequence. For removal of D-biotin, samples were precipitated by addition of trichloroacetic acid (100%) to 25% (v/v) and incubation on ice for 1 h. Proteins were pelleted by centrifugation at 13,000 rpm for 15 min at 4°C. Protein pellets were then washed 3 times with 200 μL ice-cold acetone. Washed protein pellets were dried by vacuum centrifugation at 45°C for 5 min and then resuspended in 25 μL 6 M urea, 50 mM NH_4_HCO_3._ Samples were diluted to 0.5 M urea with 50 mM NH_4_HCO_3_ before cysteine reduction (5 mM TCEP, 30 min at 37°C) and alkylation (10 mM iodoacetamide, 30 min at 37°C in the dark). Proteins were digested overnight at 37°C by addition of 1 μg trypsin (2.5 μL Promega, sequence-grade, V5113). Trifluoroacetic acid (50%) was added to 1% (v/v) to stop the reaction, and peptides were purified using C18 microspin columns (3-30 μg, Nest Group) and resuspended in 15 μL Buffer A (acetonitrile 2%, formic acid 0.1%) containing iRT peptides for retention-time alignment (Biognosys) (Escher et al., 2012).

The peptides were analyzed on an Orbitrap Fusion Lumos mass spectrometer (Thermo Scientific, San Jose, CA) connected to an Easy-nLC 1200 (Thermo Scientific, San Jose, CA) HPLC system. Between 1 μl and 4 μl of peptide solution was separated by nano-flow liquid chromatography using a 120 min gradient from 5 to 37% buffer B in buffer A (Buffer A: 2% acetonitrile, 98% H_2_O, 0.1% formic acid; Buffer B: 80% acetonitrile, 20% H_2_O, 0.1% formic acid) on an Acclaim PepMap RSLC 75 μm x 25cm column packed with C18 particles (2 μm, 100 Å) (Thermo Scientific, San Jose, CA). The peptides were ionized using a stainless steel nano-bore emitter (#ES542; Thermo Scientific) using 2000 V. The data independent acquisition (DIA) method consisted of a precursor scan followed by product ion scans using 40 windows between 400 m/z and 1000 m/z. The precursor scan was an Orbitrap full MS scan (120,000 resolution, 2 × 10^5^ AGC target, 100 ms maximum injection, 350-1500 m/z, profile mode). The product ion scans were performed using Quadrupole isolation and HCD activation using 27% HCD Collision Energy. The Orbitrap was used at 30,000 resolution using a scan range between 200 and 1800 and setting the RF Lens at 40%. The AGC Target was set to 5 × 10^5^ and 50 ms maximum injection time.

#### Data-dependent mass spectrometry

To acquire mass spectra for building a spectral library for SWATH data analysis, the mass spectrometer was operated in data-dependent acquisition mode. The mass spectrometer performed first a full MS scan (120,000 resolution, 2 × 10^5^ AGC target, 100 ms maximum injection, 350-1500 m/z, profile mode), followed by data-dependent acquisition of MS2 spectra using a duty cycle of 3 s for the most intense precursors using a 30 s dynamic exclusion time and the following HCD scan settings: 30,000 resolution, 8 × 10^4^ AGC target, 50 ms maximum injection, profile mode intensity threshold of 5 × 10^4^, charge states from 2 to 5, isolation window of 1.2 m/z using the quadrupole isolation, fragmentation with 27% HCD Collision Energy.

#### SWATH assay library generation

The raw data was analyzed using MaxQuant version 1.5.2.8 against a FASTA file containing 20,386 reviewed human or 16,985 mouse protein sequences (downloaded on August 13, 2018 from www.uniprot.org) and iRT peptides and enzyme sequences. Carbamidomethyl was defined as a fixed modification, and Oxidation (M) and Phosphorylation (STY) as variable modifications. Standard MaxQuant settings for Orbitrap were used (e.g., peptide tolerance 20 ppm for first search and 4.5 for main search). In total, three searches were performed involving 24 (mouse PD-1^OST^ series), 10 (mouse BTLA^OST^ series), 16 (human PD-1^OST^ series) and 12 (human SILAC-labeled PD-1^OST^ series) injections of unfractionated peptides and they resulted in the identification of 11,473, 3,285, 13,929, and 13,842 peptides, respectively. The four searches were imported into Spectronaut Pulsar (Biognosys, Schlieren) to build three spectral libraries with the following settings: PSM FDR Cut off of 0.01, fragment m/z between 200 and 1,800, peptides with at least 3 amino acids, fragment ions with a relative intensity of at least 5, precursors with at least 5 fragments. Moreover, an iRT calibration was performed with a minimum root mean square error of 0.8 and segmented regression. For the SILAC experiment b-ions were excluded and assays in the other channels were created *in silico* using the “label” workflow. The four spectral libraries contained coordinates for 1,310 protein groups and 9,269 proteotypic peptides (mouse PD-1^OST^), 469 protein groups and 2,279 proteotypic peptides (mouse BTLA^OST^), 1730 protein groups and 12,077 proteotypic peptides (human PD-1^OST^), and 1,840 protein groups, and 11,346 proteotypic peptides (human SILAC labeled PD-1^OST^).

#### SWATH-MS targeted data extraction

Quantitative data were extracted from the acquired SWATH-MS maps using Spectronaut Pulsar Professional+ (Biognosys, Schlieren) (version 11.0.15038.23.25164 and version 12.0.20491.13.20682 for SILAC experiment) and standard settings (they include a dynamic MS1 and MS2 mass tolerance strategy, a dynamic XIC RT Extraction Window with a non-linear iRT calibration strategy, and identification was performed using a precursor and protein Q value cutoff of 0.01). The quantified intensities for each fragment were extracted from 24 (mouse PD-1^OST^), 30 (mouse BTLA^OST^), 48 (human PD-1^OST^), and 9 (human SILAC-labeled PD-1^OST^) SWATH-MS injections and the fragment intensities were exported for further statistical analysis to R. Only quantities for fragments that have been detected at least two times in a given condition were selected. Further filtering was performed with mapDIA where a standard deviation factor of 2 and a minimal correlation of 0.25 were used to filter for reliable fragments. To avoid regulated phosphopeptides to be removed in this step, the phosphopeptides were assigned to their individual protein identifier. In the SILAC-labeled samples different protein identifiers for the two different channels were used.

#### Filtering strategy for the identification of the PD-1 and BTLA high-confidence interactomes

The quantitative values for proteins and peptides as exported by mapDIA (Teo et al., 2015) were imported into R and any missing values were imputed with a value corresponding to the 0.01 quantile intensity value for that protein or peptide across all measurements. In a first step, the proteins that significantly interact with the bait were identified by calculating their differential expression in conditions expressing the Twin-Strep-tagged bait (PD-1-OST or BTLA-OST) as compared to conditions expressing the same levels of the corresponding untagged proteins (PD-1 and BTLA). A paired and reproducibility optimized test strategy was used from the R/Bioconductor package PECA (Suomi and Elo, 2017). Proteins that showed a more than 3-fold (mouse CD4^+^ cells) or 6-fold (human Jurkat cells) higher abundance in the samples containing the bait versus control samples and an adjusted FDR of less than 0.01 across the 3 biological replicates of an experimental condition were considered as interactors. Moreover, such interactors were further filtered on the basis of their presence in at least two independent conditions and those present in the contaminant repository for affinity purification (the CRAPome ; (Mellacheruvu et al., 2013)) were eliminated. In total, 11 proteins were removed because of their presence in the CRAPome: EEF1A1, GAPDH, HSP90AB1, HSPA5, HSPA8, KRT33B, TUBA1A, TUBA1B, TUBB, TUBA4A, TUBB4B. Furthermore, any keratins, myosins, and tubulins were not considered as interactors as they correspond to highly abundant proteins that frequently appear as AP-MS contaminants. As a result, 243 (mouse PD-1^OST^), 12 (mouse BTLA^OST^), and 642 (human PD-1^OST^) interactors were significantly enriched according to those criteria. In a second step, we identified the interactors that change in abundance between the different conditions and these are considered “dynamic” interactors. To identify dynamic interactors, we normalized the protein quantities to the signal of the bait for each condition and then tested with a paired and reproducibility optimized test statistic (see above) which proteins changed in abundance between the non-stimulated (0 min time point, wild-type Raji, or no SEE) and the stimulated (time points > 0 min, PD-L1^+^ Raji cells, SEE-incubated PD-L1^+^ Raji cells) conditions. Proteins for which the signal changed on average at least 2-fold (FDR < 0.05) across the biological replicates were considered dynamic interactors. Accordingly, 12 (mouse PD-1^OST^), 5 (mouse BTLA^OST^), and 58 (human PD-1^OST^) dynamic interactors were identified in our study. Absolute abundances of the interactors were estimated with the IBAQ approach implemented in the aLFQ package (Rosenberg et al., 2014) and normalized to the bait.

#### Data analysis of SILAC-labeled AP-MS samples

The intensity values for each protein from the mapDIA software were used. To calculate the ratio, the intensity for the protein in the light channel was divided by the intensity in the heavy channel. Channels where no peak was detected a value of 8.6e02 was assigned which corresponded to the 1% lowest value detected.

### Quantification and Statistical Analysis

In all experiments, data are presented as mean ± SEM unless stated otherwise. Statistical tests were selected based on appropriate assumptions with respect to data distribution and variance characteristics. The number of biological replicates and mice is defined in the figure legends. The statistical analysis used for the identification of the PD-1 and BTLA high-confidence interactors is developed in the paragraph ‘Filtering strategy for the identification of the PD-1 and BTLA high-confidence interactomes’.

### Data and Software Availability

The MS data which were used to generate the SWATH spectral library, the SWATH raw files and the quantitative results from the SWATH-MS analysis reported in this paper have been deposited in the PRIDE proteomics data repository (https://www.ebi.ac.uk/pride/archive/) under accession numbers: PXD010862 (PD-1 mouse), PXD010863 (BTLA mouse), PXD010864 (PD-1 Jurkat), and PXD013531 (SILAC-labeled PD-1 Jurkat).

## Supplemental Information

Document S1. Figures S1–S4

Table S1. Protein and Peptide Abundance Data, Related to Figures 1, 2, and 4 Absolute label-free quantification estimates. Absolute abundances were estimated using the iBAQ approach implemented in the aLFQ package (Rosenberger et al., 2014). See STAR Methods for details.

Table S2. List of Proteins that Interacted with the Bait and that Passed the Two Applied Filters, Related to Figures 1, 2, and 4 Interactors are significantly enriched in the samples with the bait as opposed to control samples. Dynamic interactors significantly change their relative abundance as compared to the bait after stimulation. See STAR Methods for details.

Table S3. iBAQ Values, Related to Figures 1, 2, and 4

Table S4. SILAC-Based Analysis of the Jurkat or Raji Cell Origin of the Quantitatively Major Components of the PD-1 Signalosome, Related to Figures 4 and S4 See STAR Methods for details.

Document S2. Article plus Supplemental Information
