# Supplementary material for: Quantitative Interactomics in Primary T Cells Provides a Rationale for Concomitant PD-1 and BTLA Coinhibitor Blockade in Cancer Immunotherapy
Source: Cell Rep. 2019 Jun 11;27(11):3315–3330.e7. doi: 10.1016/j.celrep.2019.05.041 (PMC6581740; doi:10.1016/j.celrep.2019.05.041)
Supplement: Document S1. Figures S1–S4 [file mmc1.pdf]

Cell Reports, Volume *27*

Supplemental Information

Quantitative Interactomics in Primary T Cells Provides a Rationale for Concomitant PD-1 and BTLA Coinhibitor Blockade in Cancer Immunotherapy

Javier Celis-Gutierrez, Peter Blattmann, Yunhao Zhai, Nicolas Jarmuzynski, Kilian Ruminski, Claude Grégoire, Youcef Ounoughene, Frédéric Fiore, Ruedi Aebersold, Romain Roncagalli, Matthias Gstaiger, and Bernard Malissen

Supplemental Information

Figure S1 to S4

Excel file Table S1: Protein and peptide abundance data

Excel file Table S2: List of proteins that interact with the bait and passed the two applied filters

Excel file Table S3: iBAQ values

Excel file Table S4: SILAC-based analysis of the Jurkat- or Raji-cell origin of the components of the PD-1 interactome

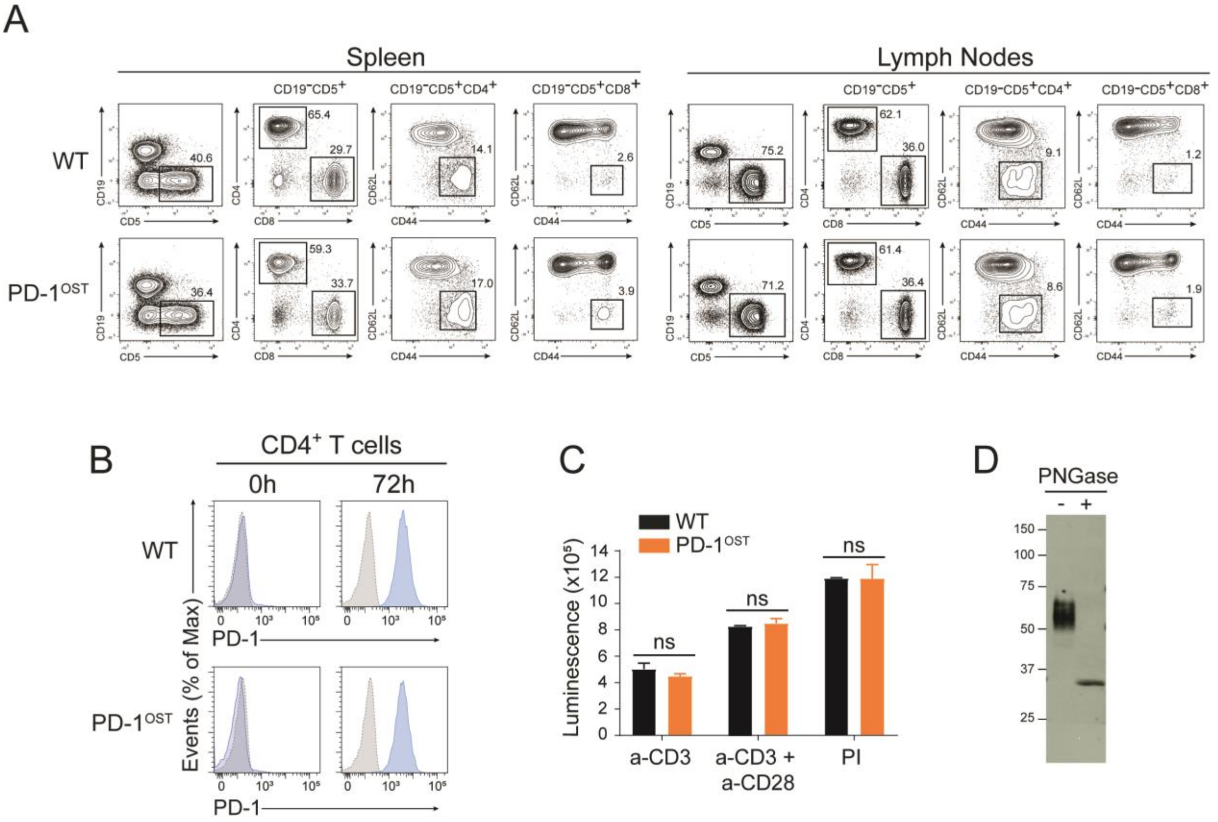

Figure S1: PD-1^{OST} mice contain normal T cells that show normal PD-1 induction upon activation. Related to Figure 1.

(A) The spleen and lymph nodes of wild-type (WT) and PD-1^{OST} mice were analyzed by flow cytometry for the expression of CD5 and CD19. CD19^{-}CD5^{+} T cells were analyzed for CD4 and CD8 expression, and CD4^{+} and CD8^{+} T cells were further analyzed for the expression of CD44 and CD62L. The spleen and lymph nodes of PD-1^{OST} mice contained normal numbers of CD4^{+} and CD8^{+} T cells with a ratio of naive (CD44^{+}CD62L^{high}) to effector-memory (CD44^{high}CD62L^{low}) cells comparable to that of WT mice. Numbers indicate the percentage of specified cells. Data are representative of at least three experiments with three mice per group.

(B) Purified CD4^{+} T cells from WT and PD-1^{OST} mice were left unstimulated (0 h) or stimulated (72 h) with plate bound anti-CD3 (145-2C11) and soluble anti-CD28 (37.51) antibodies. After 72 h of culture, T cells were harvested and stained with anti-CD4 and anti-PD-1 antibodies and CD4^{+} T cells were analyzed by flow cytometry. Numbers indicate the percentage of PD-1^{+} cells. Grey shaded curves, isotype-matched control antibody (negative control). Data are representative of at least two experiments with three mice per group.

(C) ATP content of negatively purified $CD4^+$ T cells from WT and PD-1^{OST} mice that were activated for 48 h *in vitro* with plate-bound anti-CD3 antibody in absence (a-CD3), or presence (a-CD3 + a-CD28) of soluble anti-CD28 antibody, or with PMA and ionomycin (PI). ATP content was assessed by luminescence as a measure of the extent of cell proliferation. Data are representative of at least two experiments with three mice per group (mean and SEM are shown; ns, non-significant).

(D) Purified T cells from PD-1^{OST} mice were activated with anti-CD3 and anti-CD28 antibodies for 72 h to induce PD-1 expression. Total lysates were subjected to affinity purification on Strep-Tactin Sepharose beads, followed by elution of proteins with D-biotin. Eluted proteins were incubated for 1 h at 37°C in presence (+) or absence (-) of PNGase F. Proteins were then analyzed by immunoblot with an anti-PD-1 Ab (RMP1-30). Left margin: molecular size in kilodaltons (kDa). Consistent with the presence of four N-glycosylation sites in PD-1, treatment of PD-1-OST molecules with PNGase F converted the native PD-1-OST band into a band of approximately 35 kD that corresponded to the expected molecular weight of unmodified PD-1-OST polypeptides. Data are representative of at least two independent experiments.

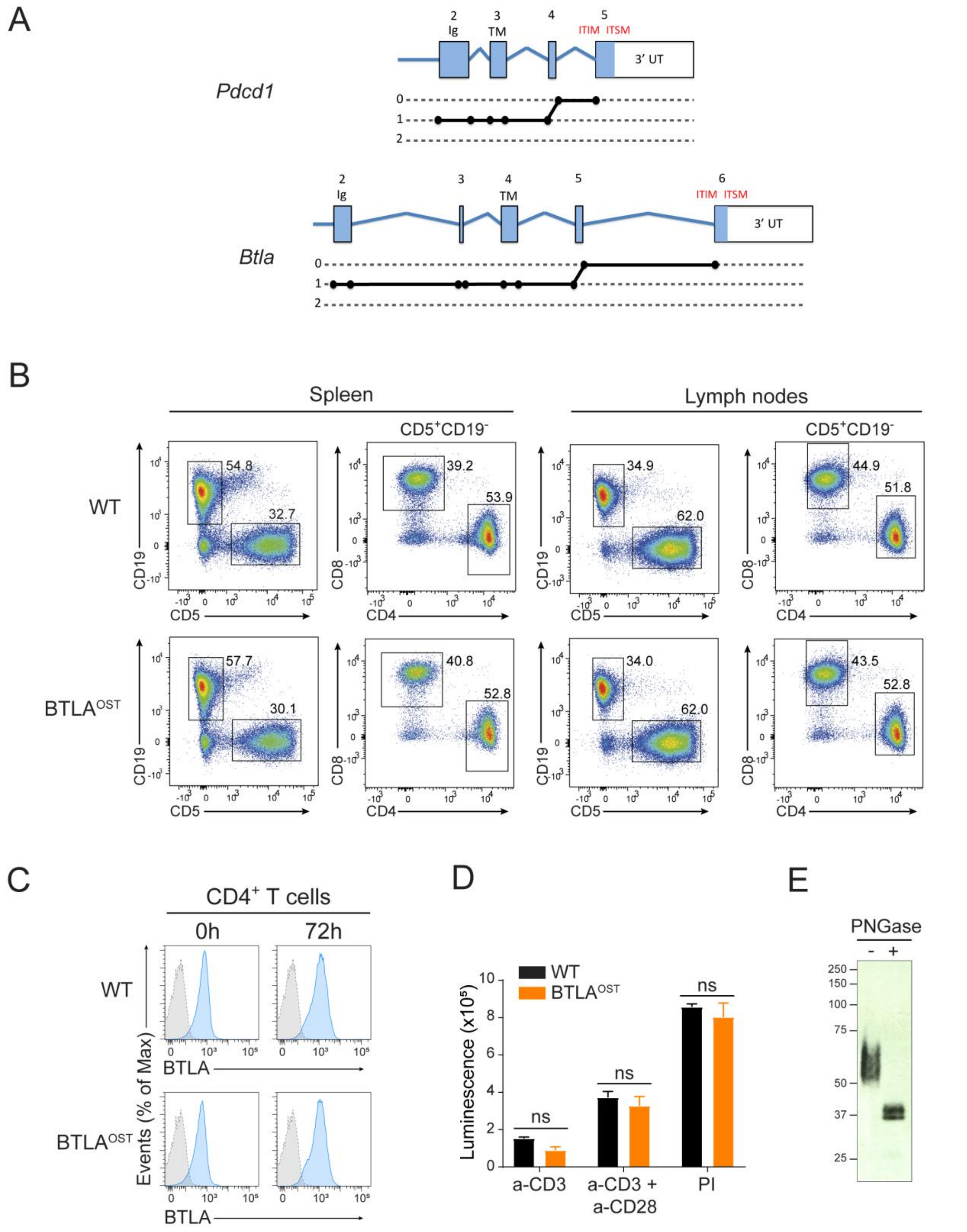

Figure S2: BTLA^{OST} mice contain normal T cells that show a normal BTLA expression. Related to Figure 2.

(A) Comparison of the exon-intron organization and of the splice frame diagrams of the mouse *Pdcd1* and *Btla* genes showed that the DNA sequences coding for the transmembrane and cytoplasmic segment of PD-1 and BTLA

display a similar exon-intron organization, that differs from that of CD28, CTLA4 and ICOS. Exons are shown as blue boxes. Based on splice frame junctions, three types of introns can be distinguished in a given gene: phase 0 intron interrupts the reading frame between two consecutive codons, whereas phase 1 and phase 2 introns interrupt the reading frame between the first and the second nucleotide of a codon or between the second and the third nucleotide of a codon, respectively. The phase class of each intron is indicated by a solid circle on the diagram shown below each gene. Ig, Immunoglobulin domain; TM, transmembrane segment; ITIM and ITSM, signaling motifs present in the cytolasmic segment, 3' UT, 3' untranslated region.

(B). The spleen and lymph nodes of wild-type (WT) and $BTLA^{OST}$ mice were analyzed by flow cytometry as described in Figure S1A.

(C) Purified $CD4^+$ T cells from WT and $BTLA^{OST}$ mice were left unstimulated (0 h) or stimulated with plate bound anti-CD3 (145-2C11) and soluble anti-CD28 (37.51) antibodies. After 72 h of culture, T cells were harvested and stained with anti-CD4 and anti-BTLA antibodies and $CD4^+$ T cells analyzed by flow cytometry. Numbers indicate the percentage of $BTLA^+$ cells. Grey shaded curves, isotype-matched control antibody (negative control). Data are representative of at least two experiments with three mice per group.

(D) ATP content of negatively purified $CD4^+$ T cells from lymph nodes of wild-type (WT) and $BTLA^{OST}$ mice activated for 48 h *in vitro* with plate-bound anti-CD3 antibody in absence (a-CD3), or presence (a-CD3 + a-CD28) of soluble anti-CD28 antibody, or with PMA and ionomycin (PI). ATP content was assessed by luminescence as a measure of the extent of cell proliferation. Data are representative of at least two experiments with three mice per group (mean and SEM are shown; ns, non-significant).

(E) Purified T cells from $BTLA^{OST}$ mice were activated with plate-bound anti-CD3 and soluble anti-CD28 antibodies for 72 h to induce BTLA expression. Total lysates were subjected to affinity purification on Strep-Tactin Sepharose beads, followed by elution of proteins with D-biotin. Eluted proteins were incubated for 1 h at 37°C in presence (+) or absence (-) of PNGase F. Proteins were then analyzed by immunoblot with an anti-BTLA antibody. Left margin: molecular size in kilodaltons (kDa). Data are representative of at least two independent experiments.

A

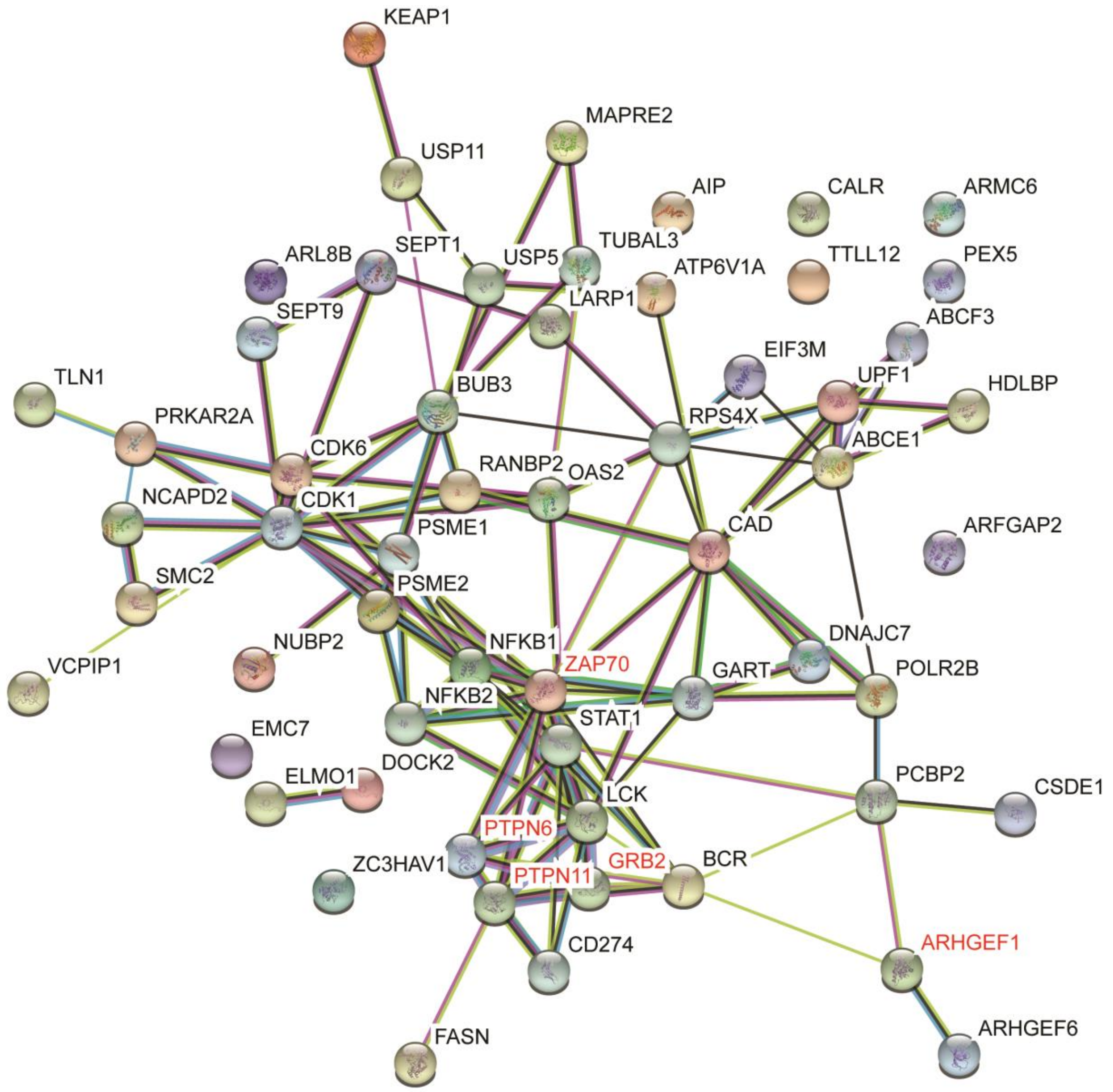

B

number of nodes:	58
number of edges:	116
average node degree:	4
average local clustering coefficient:	0.471
expected number of edges:	67
PPI enrichment p-value:	4.44E-08

Figure S3: Analysis of the human PD-1 signalosome formed at T cell-APC interface. Related to Figure 4.

(A) The dynamic PD-1 interactors identified at the T-APC interface displayed a high degree of interconnectivity compared to random proteins (p-value: 4.44e-08) and a significant enrichment for proteins related to T cell signaling and immune response (CD274, GRB2, LCK, PTPN11, PTPN6, ELMO1, NFKB1, NFKB2, ZAP70,

CDK1, OAS2, PCBP2, PRKAR2A, STAT1, ZC3HAV1). Proteins in red also interacted with PD-1 and were regulated in mouse $CD4^{+}$ T cells upon pervanadate stimulation. Among the candidate interactors whose function has not been linked to T cell activation, we found RHO/RAC guanine nucleotide exchange factors (ARHGEF 1, 2 and 6, DOCK2), enzymes involved in ubiquitin and SUMO processing (USP5, USP11, RANBP2, MAPRE2), a molecule involved in intracellular retrograde motility of vesicles along microtubules (DYNC1H1) and a protein involved in cell-cell contact (TLN1). As shown in Figure 4F, they have a low-stoichiometry of association that increased at 5 min of stimulation. As discussed in Results, they may contribute to the degradation of PD-1 molecules or allow the catalytic inactivation of associated SHP-2 molecules via reactive oxygen species. The PD-1-SHP-2 and PD-1-SHP-1 interactions are likely direct whereas the presence of GRB-2 likely results from its ability to associate with phosphorylated tyrosines found at the carboxy-terminal end of SHP-1 and SHP-2.

(B) Statistical results of network and protein-protein interactions compared to general proteome. Statistical results and network were generated using the STRING database (https://string-db.org).

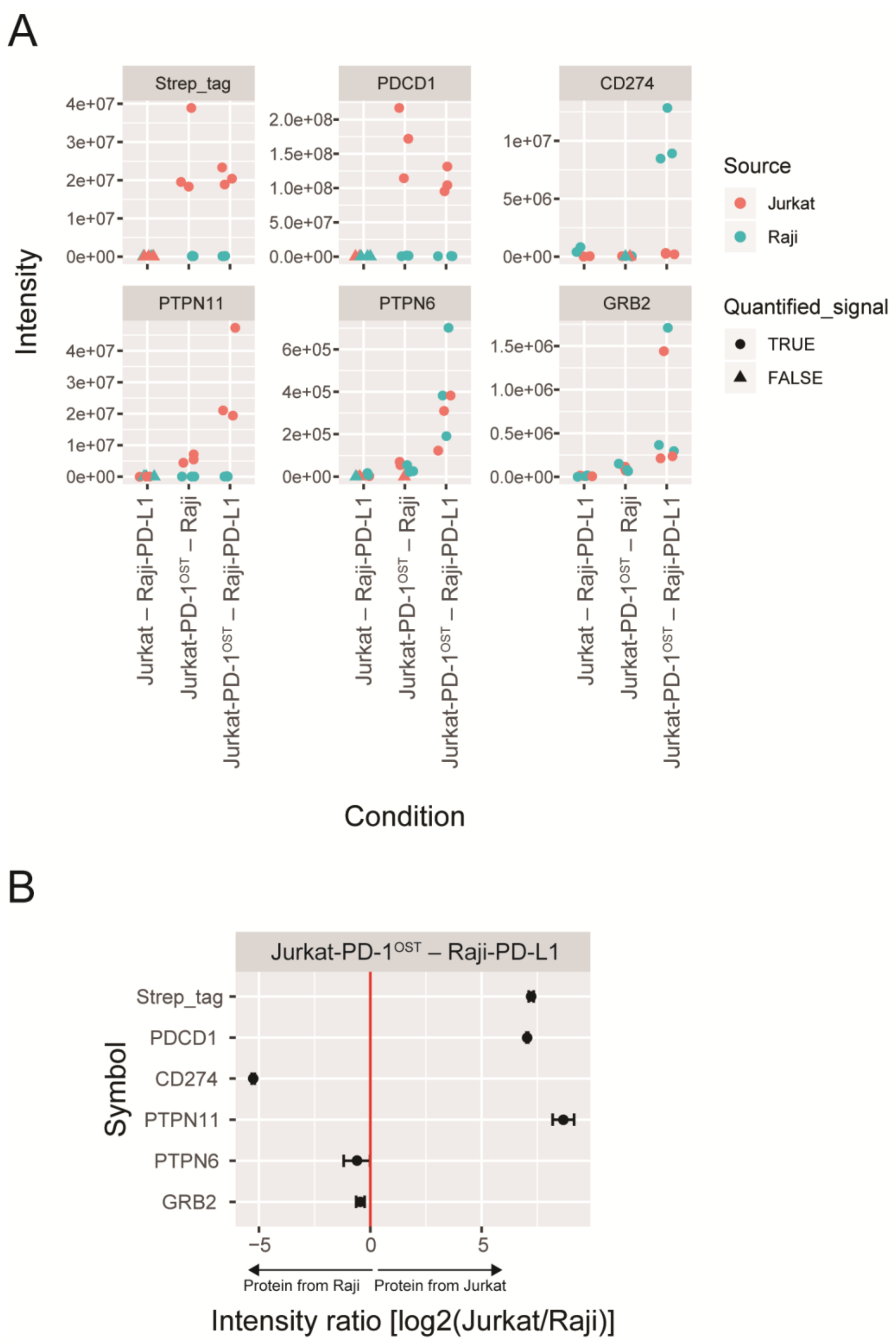

Figure S4: SILAC-based analysis of the Jurkat- or Raji-cell origin of the quantitatively major components of the PD-1 signalosome that forms at the T-APC interface. Related to Figure 4.

(A) Intensity for selected interactors across three different replicates and conditions after affinity purification on Strep-Tactin Sepharose beads. SILAC labeling of Raji cells allowed to distinguish if the protein originated from Jurkat (red) or Raji (blue) cells. If no signal was detected, the intensity is marked as a triangle.

(B) Ratio of intensity between the two different isotopically labelled proteins are shown. Mean ratio ± standard deviation are depicted for the three replicates.
